# Tailoring biomaterials for skin anti-aging

**DOI:** 10.1016/j.mtbio.2024.101210

**Published:** 2024-08-28

**Authors:** Xin Dan, Songjie Li, Han Chen, Ping Xue, Bo Liu, Yikun Ju, Lanjie Lei, Yang Li, Xing Fan

**Affiliations:** a Department of Plastic and Reconstructive Surgery, Xijing Hospital, Fourth Military Medical University, Xi'an, 710032, China; b Department of Plastic and Aesthetic (Burn) Surgery, The Second Xiangya Hospital, Central South University, Changsha, 410011, China; c Key Laboratory of Artificial Organs and Computational Medicine in Zhejiang Province, Institute of Translational Medicine, Zhejiang Shuren University, Hangzhou, 310015, China

**Keywords:** Biomaterials, Skin anti-aging, Plastic surgery, Tissue regeneration, Medical aesthetics

## Abstract

Skin aging is the phenomenon of degenerative changes in the structure and function of skin tissues over time and is manifested by a gradual loss of skin elasticity and firmness, an increased number of wrinkles, and hyperpigmentation. Skin anti-aging refers to a reduction in the skin aging phenomenon through medical cosmetic technologies. In recent years, new biomaterials have been continuously developed for improving the appearance of the skin through mechanical tissue filling, regulating collagen synthesis and degradation, inhibiting pigmentation, and repairing the skin barrier. This review summarizes the mechanisms associated with skin aging, describes the biomaterials that are commonly used in medical aesthetics and their possible modes of action, and discusses the application strategies of biomaterials in this area. Moreover, the synergistic effects of such biomaterials and other active ingredients, such as stem cells, exosomes, growth factors, and antioxidants, on tissue regeneration and anti-aging are evaluated. Finally, the possible challenges and development prospects of biomaterials in the field of anti-aging are discussed, and novel ideas for future innovations in this area are summarized.

## Graphical abstract

**Figure:**
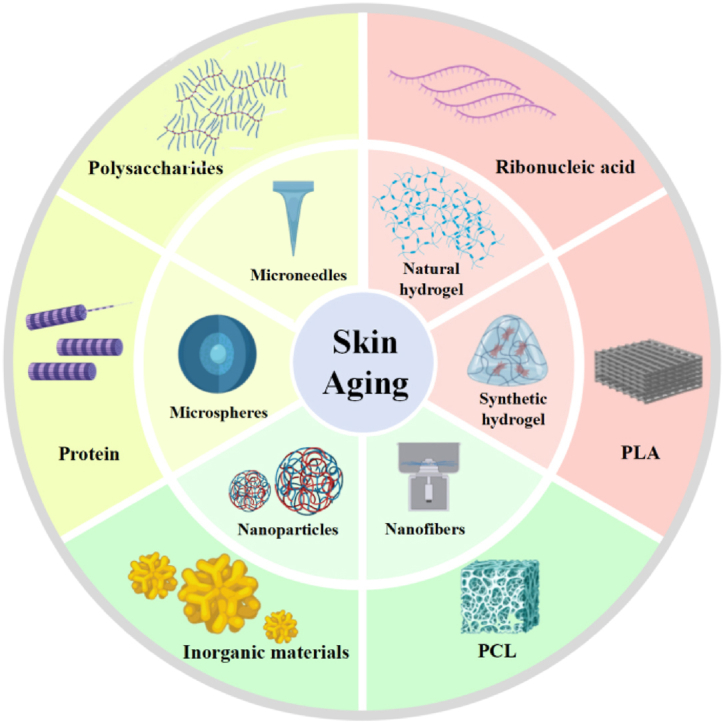

## 1 Introduction

Due to the increasing desire for beauty, the medical aesthetics industry has developed rapidly, with skin anti-aging being a key research area. Skin aging involves many changes that occur due to a combination of endogenous factors (genetic mutations, cellular metabolism, and hormonal factors) and exogenous factors (ultraviolet [UV] rays, pollutants, chemicals, and toxins). These changes are mainly due to the loss of collagen and elastin fibers, leading to a reduction in the thickness and elasticity of the dermis, decrease in the water content of the skin, slowing of the metabolism of the epidermis, and decrease in the clearance of neuroglial cells. During skin aging, the extracellular matrix (ECM) is degraded and its regenerative capacity is reduced [1]. Consequently, the skin loses its original elasticity and luster, combined with the appearance of wrinkles, pigmentation, laxity, large pores and other changes [2]. These degenerative changes not only affect skin aesthetics but can also be detrimental to the physical and mental health of an individual. According to statistics, approximately 90 % of Chinese women partake in anti-aging measures, such as skincare routines and aesthetic treatments [3].

Currently, scientific research on skin anti-aging has focused on antioxidants, cytokines, and cutting-edge technologies. Antioxidant research is dedicated to slowing the rate of skin aging by neutralizing harmful components such as free radicals [4]. Focusing on biomolecules and their function in cell signaling, cytokine research explores the mechanisms that regulate cell proliferation and differentiation, which in turn enhances skin texture and elasticity [5]. Meanwhile, technologies such as gene editing and cellular therapies are emerging as research hotspots [6]. These technologies seek to reverse the signs of aging at its root by advancing to the molecular level of skin aging. One such technology, gene editing, holds the promise of treating or preventing hereditary diseases associated with skin aging by precisely modifying genetic information [6]. Cellular therapy, on the other hand, restores the youthfulness of the skin by introducing active cells that promote the repair and regeneration of damaged tissues [7]. Despite significant progress in this area, several challenges remain, including effective selection of appropriate antioxidant types and concentrations, limited understanding of the regulatory mechanisms associated with cytokines and signal transduction, unknown safety and efficacy of gene editing, and unpredictable and uncontrollable cutaneous anti-aging effects of such treatments due to individual differences and environmental factors [8].

Recently, various new biomaterials have been developed and applied in the anti-aging field, with the aim of reducing skin aging by mechanical filling of tissues, promoting collagen regeneration, inhibiting collagen degradation, reducing hyperpigmentation, and repairing the skin barrier through drug delivery [9]. Consequently, biomaterials have become a key focus of research and market interest in recent years. Various biomaterials suitable for use in medical aesthetics have already been introduced, such as those based on recombinant collagen, HA, and polylactic acid (PLA). Other biomaterials with potential for anti-aging applications include silk proteins, mussel proteins, alginates, and bioceramics, which can be implanted in skin tissue without triggering immune or inflammatory reactions [10]. More specifically, the unique physicochemical properties and excellent biocompatibilities of such biomaterials in the form of microspheres, microneedles (MNs), hydrogels, or nanoparticles (NPs) facilitate enhanced penetration into skin tissue [11]. Consequently, they demonstrate great potential for development and application in the field of medical and aesthetic materials [12]. Furthermore, several studies have reported that biomaterials can be used as carriers of stem cells, exosomes, growth factors, and antioxidants to accelerate tissue regeneration without promoting an inflammatory response, thus demonstrating their high research value and market prospects in the field of anti-aging field of anti-aging skin [13].

This review summarizes the skin aging mechanisms and describes biomaterials commonly used in skin anti-aging techniques and their possible modes of action. Moreover, application strategies involving biomaterials in the skin anti-aging field are discussed, in addition to the synergistic effects of these biomaterials on tissue regeneration and anti-aging when combined with other active ingredients (e.g., stem cells, exosomes, growth factors, and antioxidants). Finally, the possible challenges and prospects associated with the application of biomaterials in this field are discussed, providing new ideas for the development of novel biomaterials for anti-aging applications.

## 2 skin aging

### 2.1 Mechanisms of skin aging

#### 2.1.1 Cellular aging

Cellular aging represents a microscopic aspect of skin aging and encompasses various cellular processes. Mitochondria, in particular, are pivotal in regulating cellular energy metabolism, with their dysfunction being linked to diseases that produce skin aging characteristics [14]. For instance, the POLG^D257A^ mouse model displays characteristics resembling aged skin, such as the buildup of senescent keratinocytes in the epidermis [15]. Mitochondrial damage is known to increase with age and exposure to UV radiation. This damage leads to so-called “reverse mitochondrial signaling,” which affects the synthesis and degradation of the ECM, as well as the inflammatory responses, ultimately altering the appearance of the skin [16]. Autophagy is an intracellular process that degrades and recycles the discarded organelles and dysfunctional cellular components. As noted in previous research into conditions such as progeria, as an individual ages, the efficiency of autophagy, and specifically mitochondrial autophagy (mitophagy), decreases [17]. This decline may contribute to tissue dysfunction and a diminished capacity to maintain redox homeostasis. In addition, autophagy-deficient systems typically exhibit signs of aging and cellular senescence, suggesting that autophagy possesses an anti-aging function [18].

#### 2.1.2 DNA damage

A well-established link is known to exist between DNA damage and skin aging. The skin is the largest organ of the human body and is permanently exposed to a variety of external environmental stresses, especially UV radiation, which is one of the main factors leading to skin aging and cancer [19]. UV radiation is capable of causing many forms of DNA damage, such as cross-links, base modifications, and single-strand breaks. Cells therefore trigger DNA damage response (DDR) mechanisms to uphold the genomic stability and thwart carcinogenesis [20]. These mechanisms include DNA repair, apoptosis, cell cycle checkpoints, and cellular senescence. More specifically, cell cycle checkpoints play a vital role as surveillance systems in cells to safeguard genomic integrity. When these checkpoints detect irregular chromosomal DNA structures, they block the cycle by orchestrating DNA repair processes and inhibiting the function of cell cycle regulators [21]. However, the checkpoint response also enforces cellular senescence when the cell senses unrepairable or widespread chromosomal abnormalities; although the cell remains metabolically active, it is functionally impaired and no longer undergoes mitosis. The accumulated senescent cells in the skin can lead to deleterious consequences such as skin aging [22], and so photoaging is considered a phenotypic feature of one of the main mechanisms against skin carcinogenesis. In addition, DNA damage-induced chromatin modifications, such as the formation of γ-H2AX, provide a platform to recruit downstream signaling kinase complexes and DDR proteins, which are essential for DNA repair and signaling. Notably, the ATR-Chk1 signaling pathway has an important role in skin cancer and photoaging. Activation of ATR kinase and subsequent phosphorylation of Chk1 kinase is essential for cell cycle arrest and repair upon DNA damage. However, aberrant activation of the ATR-Chk1 signaling pathway leads to permanent cell cycle arrest, i.e. cellular senescence, which may accelerate the process of tissue aging [23].

#### 2.1.3 Alterations in ECM components

During skin aging, collagen fibers undergo significant quantitative and structural changes, and these changes are the molecular basis for the appearance of wrinkles and loss of skin elasticity. Collagen is the main structural protein of the dermal ECM, exhibiting a tightly spaced and ordered network structure in young skin [24]. However, over time, collagen fibers fragment and become unevenly distributed, leading to a decrease in their overall quantity [25]. This is primarily caused by heightened matrix metalloproteinase (MMP) activity and impaired TGF-β signaling, which is in turn induced by reactive oxygen species (ROS). MMPs are a group of endopeptidases that break down ECM proteins, including collagen. During skin aging, MMP expression levels increase without a corresponding increase in the level of endogenous MMP inhibitors, resulting in the rate of collagen degradation exceeding that of its synthesis, thereby triggering an imbalance in the ECM. In addition, the accumulation of ROS activates mitogen-activated protein kinases (MAPKs), which in turn induce production of the active protein-1 and nuclear factor-kappa-B (NF-κB) transcription factors, which play key roles in the transcriptional regulation of MMP-1 and MMP-3 [26]. Thus, an imbalance between collagen degradation and synthesis is a key molecular event leading to the emergence of skin aging features.

Elastin is an important component of the skin's ECM and is responsible for maintaining its elasticity and resilience. Elastin undergoes significant structural remodeling and functional degradation during skin aging [27]. For example, studies have shown that in naturally aged skin, fibronectin-rich microfibrils are selectively degraded, whereas in photoaged skin, elastin fibers accumulate abnormally due to the activation of MMPs, resulting in so-called “solar elastosis.” This structural change leads to a decrease in the number of functional elastin fibers, which is closely associated with aging characteristics, such as the loss of skin elasticity and formation of wrinkles [28]. In naturally aged skin, the degradation of elastin fibers is associated with changes in specific elastin fiber-associated proteins, such as reduced expression of fibronectin-5, which affects the formation and stability of elastin fibers [27]. In photoaged skin, UVA and UVB radiation increases the activity of MMPs, especially MMP-12, which is a highly efficient elastin-degrading enzyme. Consequently, destruction of elastin fibers is further promoted [29].

**Fig. 1:**
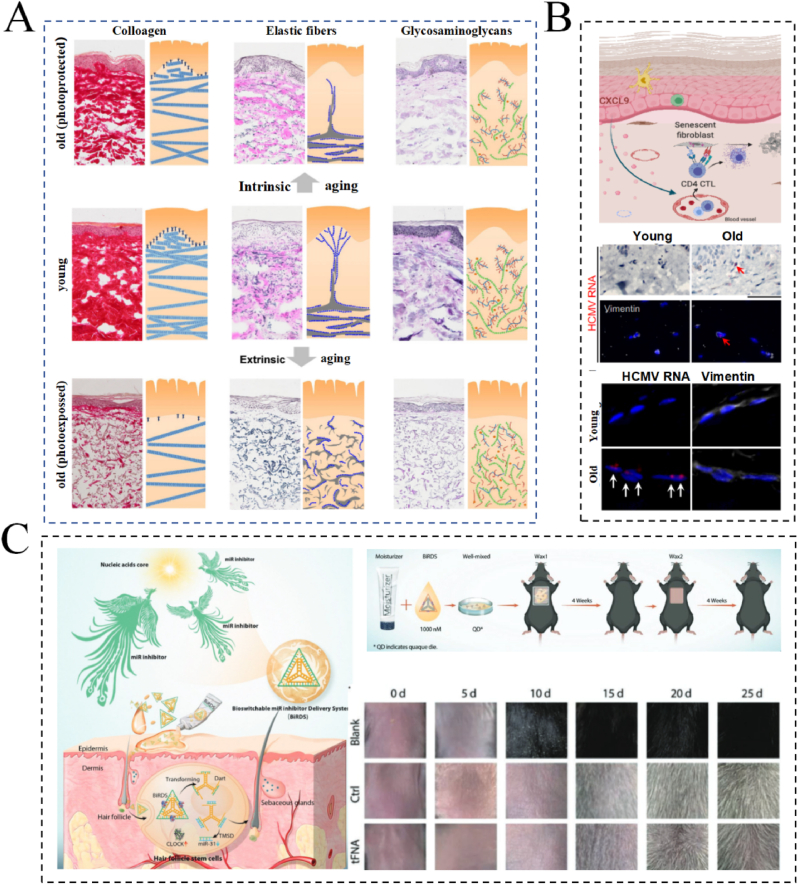
Mechanisms of skin aging and progress in anti-aging research. (A) Changes in collagen, elastic fibers, and glycosaminoglycans in normal skin, endogenous aging skin, and photoaging skin. Reprinted from Ref. [33] with permission from Elsevier. (B) One of the latest advances in skin anti-aging: cytomegalovirus immune response actively removes senescent cells. Reprinted from Ref. [34] with permission from Cell Press. (C) One of the latest advances in skin anti-aging: an innovative bioswitchable miRNA inhibitor delivery system based on tetrahedral framework DNA. Reprinted from Ref. [35] with permission from Wiley. Abbreviations: CD4 CTL, cytotoxic CD4^+^ T cells; HCMV, human cytomegalovirus; miR, microRNA/miRNA.

Proteoglycans (PG) and HA are key components of the skin's ECM and play an essential role in the maintenance of skin structure and function. During skin aging, the balance between degradation and synthesis of these ECM components changes, resulting in a decrease in skin elasticity and water retention [30]. PGs, such as decorin and biglycan, participate in the construction and stabilization of the ECM through interactions with collagen and elastin. Over time, the expression and function of these PGs may change, affecting the integrity of the ECM and altering the structural stability of the skin [31]. HA, a non-sulfated glycosaminoglycan, exhibits a unique water-binding ability and is engaged in the modulation of cell signaling and proliferation through its binding to ECM proteins. During skin aging, HA synthesis may be inhibited, whereas its degradation is promoted, leading to impaired water retention ability and reduced ECM function. In addition, HA degradation products can contribute to aging by influencing cellular behavior, such as promoting inflammation and facilitating fibroblast proliferation [32]. These changes suggest that the role of HA and PGs in skin aging goes beyond acting as ECM fillers; they are also involved in regulating the cellular microenvironment, affecting cellular function, and influencing the overall health of the skin (Fig. 1A) [33].

#### 2.1.4 Oxidative stress and damage

ROS are chemicals produced during cellular metabolism, and they are involved in both cell signaling and maintaining a redox balance under normal physiological conditions. However, when ROS production exceeds the scavenging capacity of the cell's internal antioxidant system, oxidative stress occurs, which is destructive to macromolecular cell components, including lipids, proteins, and nucleic acids [36]. During skin aging, ROS accumulation activates multiple signaling pathways, including the NF-κB and MAPK signaling pathways, inducing the expression and activation of MMPs [37]. Notably, aberrant activation of MMPs leads to degradation of ECM components, especially elastin fibers and collagen, which affects the skin's elasticity and integrity, resulting in the formation of aging features such as wrinkles and sagging [38]. Furthermore, ROS can directly damage ECM components and intensify the skin's aging process. For example, ROS can promote the cross-linking and glycosylation of collagen, leading to a decrease in its functionality [39]. Chronic ROS exposure may also lead to a decrease in the activity of antioxidant enzymes, such as glutathione peroxidase (GPX) and superoxide dismutase (SOD) in skin cells, further weakening the cellular defenses against oxidative stress [40].

#### 2.1.5 Inflammatory factors and chronic inflammation

Inflammatory cytokines play an important role in the process of skin aging, and their increase is closely linked to chronic inflammation. During aging, the levels of inflammatory cytokines in the skin rise; these cytokines include interleukin (IL)-1, IL-6, and interferon gamma and tumor necrosis factor alpha (TNF-α) [41], which are produced by various cells, including keratinocytes, fibroblasts, and immune cells (e.g., T cells and macrophages). Elevated levels of inflammatory cytokines activate a variety of signaling pathways such as the NF-κB and MAPK pathways, which affect the structure and function of the skin [42]. In addition, inflammatory cytokines can influence the immune response of the skin, which may lead to increased sensitivity to external stimuli, thereby exacerbating the skin aging process. For instance, IL-6 is not only involved in the inflammatory response but is also associated with the aging of skin fibroblasts, wherein it promotes degradation of the ECM, leading to loss of skin elasticity and firmness [43]. Continued production of inflammatory cytokines in a chronic inflammatory state may lead to a constant cycle of skin cell damage and repair, ultimately accelerating the appearance of aging characteristics.

### 2.2 Common anti-aging strategies

Currently, research into skin anti-aging focuses on several areas, including antioxidants, cytokines, and emerging technologies. More specifically, research into antioxidants aims at slowing the process of skin aging by scavenging harmful substances, such as free radicals. In this context, ascorbic acid is an important antioxidant that can effectively eliminate most ROS and exert antioxidant effects through oxidation and re-oxidation processes. For example, ascorbic acid is a cofactor for proline hydroxylase, which promotes the hydroxylation of proline residues in collagen and elastin to help maintain skin structure and elasticity [44]. In recent years, researchers have discovered new functions of ascorbic acid, including its ability to promote epidermal cell differentiation and skin barrier formation [45]. As another example, tocopherol, an important form of vitamin E, not only neutralizes the excess ROS produced by oxidative stress and reduce damage caused to cells, but also binds directly to cell membranes and prevents free radicals from attacking lipids and proteins within these membranes [46]. Kamei et al. compared the effects of different vitamin E analogs on melanin formation and found that certain tocopherols could effectively inhibit melanin synthesis with reduced toxicity to the skin [47]. Therefore, these analogs were proposed to help prevent or ameliorate skin hyperpigmentation, thereby slowing the UV-induced aging process of the skin. In terms of cytokines, studies into these compounds and their role in signal transduction have explored how skin texture and elasticity can be improved by regulating cell growth and differentiation. For instance, Idkowiak-Baldys et al. demonstrated that growth differentiation factor 11 promotes the proliferation of skin cells, including fibroblasts and keratin-forming cells, by inducing the phosphorylation of Smad2/3 [48]. This leads to an increase in the production of pre-collagen and HA and, consequently, to less pronounced wrinkle formation during aging. Moreover, explorations based on emerging technologies, such as gene editing and cellular therapy, aim to reverse the skin aging process at its root. For example, Hasegawa et al. found that senescent fibroblasts express human cytomegalovirus glycoprotein B antigen and human leukocyte antigen class II, which are directly targeted by cytotoxic CD4^+^ T cells. Cytotoxic CD4^+^ T cells directly damage senescent cells by recognizing human cytomegalovirus glycoprotein B antigen (Fig. 1B) [34]. Widgerow et al. developed the TriHex™-containing Restorative Skin complex Anti-Aging Formula, which significantly reduces telomere shortening, upregulates *FOXO3* and *KLOTHO*, and demonstrates favorable anti-aging properties [49]. In addition, Li et al. constructed an innovative bioswitchable miRNA inhibitor delivery system based on tetrahedral framework DNA, through which miR-31 inhibitors with anti-aging effects could be delivered to a skin aging model, and observed good skin permeability and significant RNA delivery capacity (Fig. 1C) [35].

Despite significant progress in this area, several challenges remain, including effective selection of the appropriate antioxidant type and concentration, limited knowledge of the regulatory mechanisms associated with cytokines and signal transduction, the unknown safety and efficacy of gene editing, and the unpredictability and uncontrollability of the skin anti-aging effects of such treatments owing to individual differences and environmental factors [50]. However, the development of biomaterials for application in skin anti-aging has provided new ideas for solving these problems, as described below.

## 3 General requirements for regenerative materials

### 3.1 Biocompatibility

Good biocompatibility, meaning the ability of a biomaterial to interact with a living organism without causing negative biological responses, is a fundamental principle in designing and using biomaterials for anti-aging skin treatments. A biocompatible material should be able to coexist harmoniously with human tissues without causing immune, inflammatory, or toxic reactions while also maintaining its functional and structural stability over the expected period [51]. For example, as mentioned above, HA is a biomaterial that is widely used in medical aesthetics and is favored due to its high biocompatibility and excellent moisturizing ability. HA is a naturally occurring polysaccharide in the body, and to date, its application in medical aesthetics includes dermal fillers and wrinkle improvement. Since HA is similar in composition to the body's own ECM, it is highly biocompatible and usually does not cause an immune response [52]. In addition, collagen, a key component of the skin structure, is commonly used in anti-aging skin products [53]. The biocompatibility of collagen can be mainly attributed to its natural presence in the human body, although it can also be produced using recombinant DNA technology to reduce any potential risks of immunogenicity or disease transmission [54]. For example, the recombinant humanized collagen filler “VYMIX” has been shown to possess a similar amino acid sequence and triple helix structure to that of human collagen, ensuring its biocompatibility in aesthetic applications [55]. To further improve the biocompatibilities of biomaterials, researchers have adopted various strategies, including surface modifications, chemical modifications, and composite techniques. Nanotechnology and the modification of biofunctional molecules can also be used to improve the stabilities and bioactivities of biomaterials, thereby promoting improved performances *in vivo* [56].

### 3.2 Immunogenicity

**Fig. 2:**
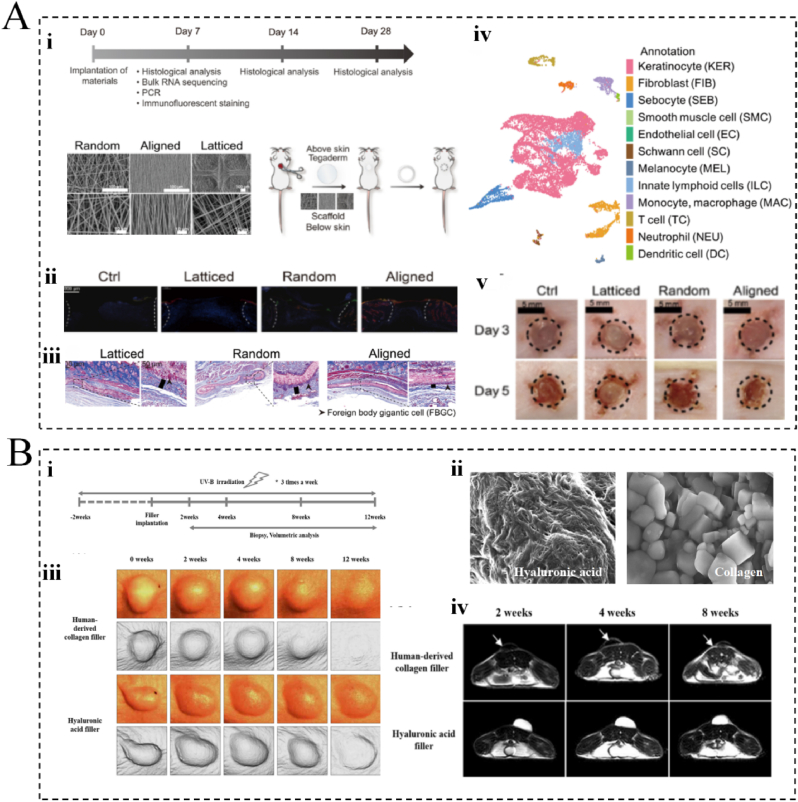
Biocompatibility and biodegradability requirements for biomaterials. (A) Electrospun membranes with three different surface topologies (random, aligned, and latticed). (*i*) Surface morphology and workflow on the mouse surface. (*ii*) Immunofluorescence staining for foreign body reactions. (*iii*) Histological staining of foreign body reactions. (*iv*) Cellular composition of foreign body reactions. *v*) Observations on the surface of mice. Reprinted from Ref. [58] with permission from the American Association for the Advancement of Science. (B) Hyaluronic acid and collagen injected into mice. (*i*) Schematic diagram of workflow. (*ii*) Schematic diagram of hyaluronic acid and collagen under electron microscopy. (*iii*) Three-dimensional simulation image of degradation rate. (*iv*) Magnetic resonance imaging performance during degradation. Reprinted from Ref. [61] with permission from Springer.

In the use of biomaterials for skin anti-aging, special requirements are placed on the immunogenicity of the material. Immunogenicity refers to the ability of a material to activate the host immune system, a property that serves a dual purpose in anti-aging skin treatments. On the one hand, a moderate inflammatory response stimulates the production of collagen by fibroblasts, which improves skin elasticity and reduces wrinkles. Conversely, an excessive immune response may lead to adverse reactions, thereby affecting treatment efficacy and patient experience (Fig. 2A) [57,58]. For example, when poly (L-lactic acid) (PLLA) particles are injected into the skin, a subclinical foreign body inflammatory response is triggered, which activates inflammatory cells such as neutrophils and macrophages. These inflammatory cells are involved in the removal of foreign body particles, in addition to releasing growth factors and cytokines to stimulate collagen production by the fibroblasts. As the PLLA particles gradually become encapsulated in fibrous tissue, they provide a stable scaffold for collagen deposition, which promotes repair and regeneration of the skin structure and enhances its elasticity and firmness. This process not only provides more pronounced results immediately following filler treatment, but it also helps to achieve long-term anti-aging effects [59]. However, the excessive inflammatory response produced by PLLA can cause a series of adverse reactions in the skin, such as redness, swelling, pain, itching, and even localized tissue necrosis, which may counteract its positive anti-aging effects [60]. Therefore, researchers and clinicians must precisely control the biomaterial dosage and injection technique, while also ensuring careful patient monitoring and follow-up care.

### 3.3 Biodegradability

The biomaterials used in skin anti-aging should be biodegradable to ensure that they are gradually absorbed and metabolized by the human body, thereby avoiding rejection and tissue damage [62]. Ideally, the degradation products should also provide support and stimulation to the damaged tissues, facilitating tissue regeneration and repair and ultimately promoting replacement by the body's own tissues (Fig. 2B) [61]. Some common biodegradable biomaterials include PLA, polycaprolactone (PCL), and gelatin. These biomaterials are often engineered to break down and be gradually absorbed in the body, and they are known to possess different degradation rates and metabolic pathways, thereby allowing their careful and precise selection based on specific therapeutic needs and desired outcomes [63]. In specific applications, biomaterials can be degraded by controlling their mechanical properties, chemical composition, structure, and preparation processes [64]. For example, the degradation rate of PLLA can be achieved in numerous ways. A common approach is to modify the surface properties of the scaffold, e.g. by surface coating or introducing other compounds to modulate its surface activity or hydrophilicity. This ultimately influences the interactions between the surrounding tissues and the scaffold to tune the rate of degradation. Another approach is to combine PLLA with other biomaterials, such as collagen polymers or gelatin, to control its degradation rate *in vivo*. The degradation rate of a biomaterial can also be influenced by modulating the pore structure and microscopic morphology of the scaffold, such as through the preparation of nanofiber scaffolds [65]. In this regard, Shamsah et al. investigated the *in vitro* degradation of 50:50 PCL/PLLA electrospun silk scaffolds over a period of 6 months [66]. They observed that this scaffold had significant hydrolytic activity and higher stiffness compared to pure PLLA and PCL scaffolds.

### 3.4 Surface physical and chemical properties

**Fig. 3:**
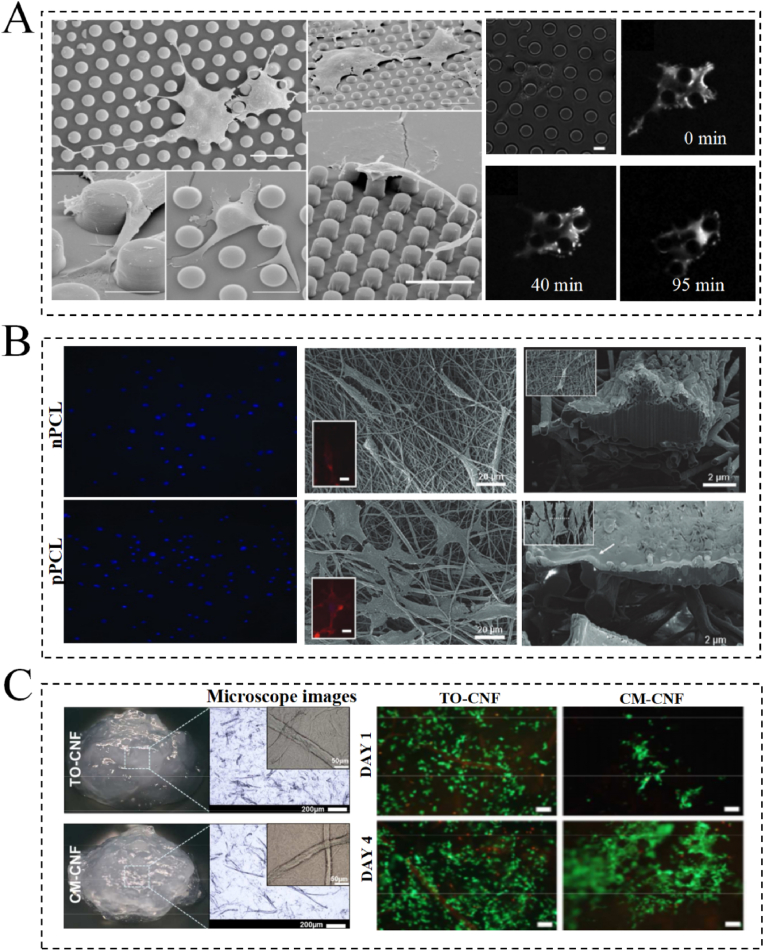
Surface physicochemical properties of biomaterials. (A) Cells exhibit different proliferation and morphologies on surfaces with different roughness levels. Reprinted from Ref. [68] with permission from Cell Press. (B) Morphology and adhesion behavior of cells on positive and negatively variable charges. Reprinted from Ref. [71] with permission from Mary Ann Liebert, Inc. (C) Biomaterials modified by different chemical functional groups have different functions for cells. Reprinted from Ref. [73] with permission from the American Chemical Society. Abbreviations: CM-, Carboxymethylated; CNF, cellulose nanofibril; nPCL, negatively charged PCL; pPCL, positively charged PCL; TO-, TEMPO oxidized.

As anti-aging research has intensified, growing evidence suggests that surface properties of biomaterials play a crucial role in regulating the behavior of skin cells, collagen, and other related cells [67]. For example, the surface roughness properties of biomaterials have been reported to significantly affect cell growth and function, with an appropriate surface roughness promoting cell adhesion and proliferation and simultaneously improving skin repair and regeneration (Fig. 3A) [68,69]. In this context, Ross et al. found that biomaterials with an average roughness (Ra) of ∼7.7–19.8 nm not only promote fibroblast proliferation and growth with collagen secretion but also enhance the adsorption of collagen [70]. This discovery is expected to be applicable to skin anti-aging due to its potential to maintain skin firmness and elasticity. In addition, the surface charge of a biomaterial plays a crucial role in determining the anti-aging behaviors of cells [67]. Typically, negatively charged surfaces both moisturize and nourish the skin and promote fibroblast adhesion and collagen growth. Positively charged surfaces, on the other hand, typically inhibit the inflammatory response of the skin while reducing cell proliferation activity (Fig. 3B) [71,72]. For example, McCarthy et al. found significant changes in cell–collagen interactions when collagen contained negatively charged cobalt ions; fluorescent images and bioassays showed that with increasing cobalt concentration, the level of cell proliferation and viability decreased, thus preventing collagen formation [72]. These results suggest that skin anti-aging can be effectively modulated by regulating the surface charges of biomaterials. Similarly, the chemical properties of the surface, such as its functional groups, chemical composition, and hydrophilicity, can also affect cell adhesion, growth, and differentiation. More specifically, surfaces containing functional groups, such as carboxyl and hydroxyl groups, can promote cell adhesion and proliferation and improve the skin repair ability (Fig. 3C) [67,73]. In this context, Rashad et al. prepared wood-derived cellulose nanofibril (CNF) hydrogels with different surface chemistries using the TEMPO oxidation and carboxymethylation methods [73]. While the carboxymethylated CNF hydrogels were able to maintain favorable cell viability, proliferation, and migration rates, they negatively impacted cell morphology and spreading. Conversely, TEMPO-oxidized hydrogels preserved the fibroblast spreading characteristics and morphology. Therefore, ideal biomaterials for anti-aging applications should be able to effectively moisturize, firm, nourish, and repair the skin, in addition to slowing the aging process. An in-depth study of the relationship between the anti-aging efficacy and surface physicochemical properties of biomaterials would therefore promote the development of targeted anti-aging products.

## 4 Commonly used regenerative biomaterials

### 4.1 Natural polymers

#### 4.1.1 Proteins

Collagen is a naturally occurring biomaterial that plays key structural roles in the skin, including supporting joint formation and enhancing tensile strength and flexibility. Type I collagen plays a key role in supporting fibroblasts and keratinocytes and controls a variety of functions including cell shape, differentiation, and migration, as well as promoting skin regeneration by facilitating the synthesis of ECM proteins in the skin. Some collagen supplements also have antioxidant properties that enhance SOD and GPX activities, thus preventing free radical damage and other ROS, while also having excellent anti-aging potential [74]. Recent studies have shown that marine-sourced collagen peptides and collagen have higher bioavailability, efficacy, and safety compared to mammalian-sourced collagen. This can be attributed to the fact that marine collagen originates from a wide range of natural sources, contains no additives, and is generally well accepted by the human body. These properties give marine collagen a wide range of applications in the fields of beauty and health care [75]. In this context, Herawati et al. identified pepsin-soluble collagen and hydrolyzed collagen (HC) on scombroid skin and demonstrated its antiglycation*,* antioxidant, and antityrosinase activities; these properties were assumed to correspond to potential anti-aging properties [76]. Furthermore, after oral administration of a hydrolyzed marine collagen supplement (Vinh Wellness Collagen), Evans et al. evaluated skin elasticity, wrinkles, and self-reported aging in a randomized triple-blind clinical trial of women aged 45–60 years using the Visual Analogue Scale of Skin Quality (VASQ) and the VISIA Skin Analysis System, Cutometer® [77]. Six weeks after treatment, the subjects exhibited a significant improvement in skin elasticity on their cheeks, while after 12 weeks, the appearance of their skin improved in terms of observed wrinkles, elasticity, hydration, radiance and firmness. These results suggest that marine collagen has great promise for research and anti-aging applications. In addition to marine collagen, human collagen-derived peptides have been the subject of a great deal of scientific attention. For example, Hwang et al. identified and described a specific segment of a protein derived from human *COL1A2* [78]. This protein encourages the growth of fibroblasts and the production of type I collagen. Their research demonstrated that a peptide derived from human collagen α-2 type I stimulated the creation of type I collagen, cell growth, movement, and elastin production, indicating notable benefits in reducing wrinkles. Their study was therefore considered to be of great significance for the clinical application of human collagen-derived peptides. Additionally, the amino acid sequence of recombinant collagen, which is prepared through bioengineering, can be designed according to the desired application. Recombinant collagen has also been demonstrated to exhibit good tissue compatibility and has become a hot topic in skin anti-aging research [64]. Furthermore, Wang et al. implanted recombinant humanized type III collagen (rhCol III) as a biological active material *in vivo* and showed that rhCol III was able to mitigate UV radiation-induced photoaging by increasing the secretion of collagen I and collagen III, decreasing the thickening of the epidermis and dermis as well as remodeling the ECM [79]. However, the technology for the preparation of recombinant collagen is still in the research and development stage and the cost is relatively high, which limits its widespread use in skin anti-aging. Therefore, further improvements can still be made to recombinant collagen, with a view to reducing costs and standardizing market applications while providing more prominent anti-aging effects and biocompatibility [80].

**Fig. 4:**
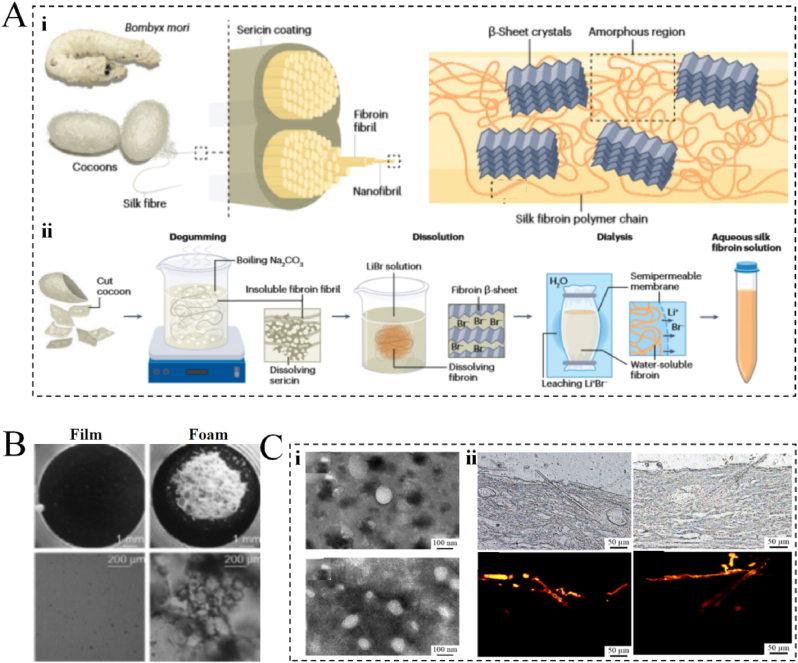
Silk protein. (A) Hierarchical structure of silk fibroin and its processing conditions. (*i*) Schematic diagram of the hierarchical structure of silk fibroin in different extraction steps. (*ii*) Extraction of silk fibroin from silkworm cocoons to produce regenerated silk fibroin solution. Reprinted from Ref. [81] with permission from Nature Portfolio. (B) Different forms of filamentous proteins. Reprinted from Ref. [83] with permission from Elsevier. (C) Penetration of silk protein nanoparticles in the skin. (*i*) Transmission electron microscopy images of silk fibroin nanoparticles and fluorescent fibroin nanoparticles. (*ii*) Fluorescence micrographs of a cross-section of mouse skin at 2 and 6 h after administration of rhodamine B solution and illumination of the same cross-section in bright and dark fields. Reprinted from Ref. [84] with permission from Elsevier.

Silk fibroin (SF) is a newly developed protein biomaterial that is prepared by processing sericin fibers after silk degumming (Fig. 4A) [81,82], and it can take various forms such as powder, film, and fiber (Fig. 4B) [83]. Because of its excellent skin penetration, good biocompatibility, degradability, minimal immunogenicity, easy availability, and low cost, SF has been widely used in the field of skin anti-aging (Fig. 4C) [84,85]. In general, silk proteins possess good supporting properties for fibroblasts and human keratinocytes, thereby promoting cell differentiation, proliferation, and adhesion, while enabling skin regeneration and repair, helping maintain healthy and youthful skin [86]. Recently, Choi et al. reported that γ-irradiated filaggrin (I-filaggrin) effectively reduced oxidative stress-induced ROS production and lowered the levels of oxidative stress-induced inflammatory factors (i.e., iNOS, COX-2, IL-1β, and TNF-α) [86]. This discovery further demonstrates the anti-aging mechanism of filaggrin proteins. SF can also be bound to other polymers, such as HA and collagen, to form SF-based composite scaffolds that synergistically promote cellular behavior, thereby enhancing anti-aging effects [87]. For example, Wani et al. found that layering SF with type I collagen enhanced the ability of keratin-forming cells to attach and disperse, thereby promoting regeneration of the stratum corneum (SC) and improving the skin's ability to combat external aging factors [88].

*Mytilus edulis* adhesive protein (MAP), a collection of natural protein-based block copolymers secreted by the pedunculated filamentous glands of the bivalve mussel *Mytilus edulis*, is known to exhibit low immunogenicity along with good biocompatibility, degradability, and adhesion properties [89]. Owing to their outstanding anti-inflammatory and antioxidant properties, mussel proteins also have a positive effect on skin aging, and consequently, MAP is commonly used in tissue engineering. In this context, Zhou et al. established an H_2_O_2_-induced MRC-5 cell model of premature senescence [90]. They isolated and identified mussel oligopeptides from mussel protein hydrolysate to investigate how mussel oligopeptides affect cellular senescence. These mussel oligopeptides were not only able to significantly increase cell proliferation and survival potential but also inhibit senescence-associated-β-galactosidase activity, thereby preventing H_2_O_2_-induced cellular senescence. In another study, Zhou et al. found that mussel peptide preparations attenuated oxidative damage, reduced ROS levels (e.g., H_2_O_2_), decreased the lipofuscin content, and delayed apoptotic cell death [91]. In recent years, due to the continuous development and application of bacterial expression systems and recombinant DNA technologies, MAPs have undergone high-yield and multifunctional modifications, providing technical support for their further development in the field of skin anti-aging [92]. More specifically, recombinant MAPs have been prepared as multifunctional nanofibrous scaffolds with enhanced mechanical strengths, biocompatibilities, and surface adhesion properties, facilitating the rapid introduction of a wide range of biomolecules and promoting the development of skin tissue engineering [93]. Furthermore, Kim et al. investigated the role of MAP hybrid nanofiber mats in promoting skin growth and found that these structures facilitated the extensive spread and rapid growth of keratinized cells, enhanced cell/tissue infiltration, promoted formation of the basal lamina, and increased growth factor encapsulation threefold [94]. Such characteristics would be expected to help deliver signals that stimulate cell proliferation, differentiation, and ECM secretion, while preserving growth factors that are inherent to the peripheral region of the skin.

#### 4.1.2 Polysaccharides

**Fig. 5:**
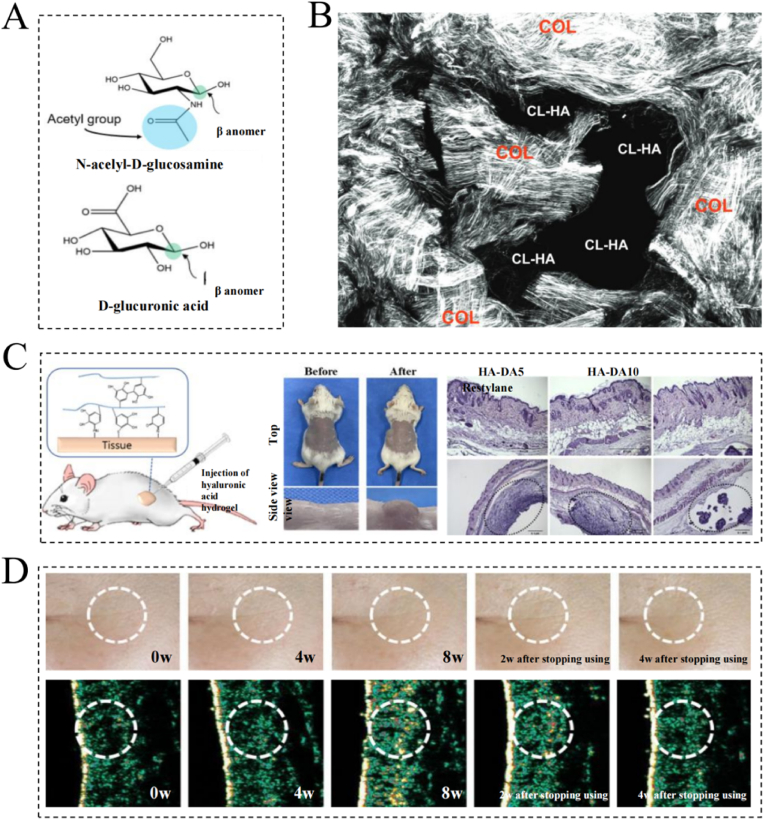
Hyaluronic acid. (A) Schematic of the chemical structure of hyaluronic acid. Reprinted from Ref. [95] with permission from Elsevier. (B) Collagen regeneration in skin tissue after injection of hyaluronic acid fillers. Reprinted from Ref. [97] with permission from Lippincott Williams & Wilkins. (C) Schematic and *in vivo* experimental performance of hyaluronic acid hydrogels as tissue fillers. Reprinted from Ref. [98] with permission from Elsevier. (D) Changes in skin wrinkles at different times after hyaluronic acid injection and after stopping injection. Reprinted from Ref. [99] with permission from Wiley. Abbreviations: CL-, cross-linked; COL, collagen; HA, hyaluronic acid.

HA is a long-chain unbranched polysaccharide composed of N-acetyl-D-glucosamine and D-glucuronic acid (Fig. 5A) [95,96]. As mentioned above, the anti-aging efficacy of HA is closely related to its ability to improve skin hydration, induce soft tissue strengthening, stimulate collagen production, and promote facial rejuvenation (Fig. 5B–D) [[97], [98], [99]]. Consequently, HA has been used in the field of skin anti-aging, and the application of this polysaccharide has been favored due to its good biodegradability and biocompatibility [52]. Importantly, a low incidence of filler complications has been demonstrated, although these can be corrected by hyaluronidase injections if an adverse event were to occur. Considering these outstanding advantages of HA in the context of anti-aging, its application techniques have received growing research attention in recent years. For example, Lee et al. developed a novel cross-linked HA patch (CLHA patch), which initially resisted wrinkle formation through the hydrating and swelling effects of HA [100]. Subsequently, this patch was found to stimulate fibroblasts and increase collagen production to offer the skin a fuller appearance [101]. Furthermore, the presence of cross-linked HA solutions in CLHA microstructured plaques could overcome the rapid turnover of native HA in the epidermis and improve its sustainability [100]. Researchers have also increased the efficacy of HA through its combination with other anti-aging substances. For example, Avadhani et al. explored co-encapsulation of HA and epigallocatechin gallate (EGCG) in a single nano-drug carrier system for synergistic anti-aging and antioxidant efficacy [102]. This system demonstrated a significant free radical scavenging capacity, low cytotoxicity, effective inhibition of MDA and ROS levels, and reduced MMP expression, thereby leading to enhanced protection against UV radiation and providing both antioxidant and anti-aging effects. However, HA-based fillers are usually prepared using chemical cross-linkers, such as divinyl sulfone and butylene glycol diglyceride ether, which have *in vivo* lifetimes of only 6 months, thereby leading to the requirement of repeated injections [103]. This use of chemical cross-linking agents and the necessity for repeated surgeries may therefore trigger adverse inflammatory and immune-mediated reactions, thereby rendering them inconvenient and expensive to perform [104]. Therefore, the development of new injectable materials that do not require chemical cross-linking is currently required.

Chitosan is a copolymer that is obtained by controlling the deacetylation of chitin [105]. Chitosan is a natural resource that exhibits excellent biodegradability [106], biotolerance [107], and antimicrobial capacity [108]. Consequently, the US Food and Drug Administration (FDA) has approved its use as a biomaterial for applications that aim to restore function to defective or lost tissues [106]. Furthermore, chitosan inhibits the activity of MMPs during skin aging, thus protecting elastin fibers and collagen and reducing both skin laxity and wrinkle formation [109]. Chitosan also exhibits good antioxidant properties and enzyme inhibitory activities (i.e., anti-hyaluronidase and antityrosinase activities), thereby reducing skin damage and promoting healthy skin [110]. In recent years, the adjustment of various parameters, such as chitin concentration, chitin/alkali ratio and temperature, has allowed the production of chitosan from the end product with a precise degree of deacetylation [111]. Specifically, re-acetylated chitosan has become a key research target because of its high-water solubility and stability. In this context, Afonso et al. reported that films prepared from re-acetylated chitosan were not only less immunogenic and more biocompatible, but also slowed the rate of antioxidant release when encapsulated, providing longer-lasting anti-aging potential [109]. The combination of chitosan with other biomaterials has also been investigated to expand its use in skin anti-aging applications [112]. For example, Libio et al. prepared a citrate buffer-neutralized chitosan membrane (with and without HA) and demonstrated that a porcine skin model treated with this membrane exhibited improved-cuticle shed, along with a significant increase in hydration in 10 min, ultimately promoting the skin exfoliation process [113]. In addition, Danti et al. combined chitosan nanofibers with nanolignin to produce a composite material with unique properties [114]. This composite both trapped hydrophilic and lipophilic molecules and served as a carrier for the delivery of various beneficial ingredients (e.g., vitamins, trace elements, anti-inflammatory agents, and antioxidants).

Alginate is an FDA-approved polymer and one of the most promising biomaterials for regenerative medicine applications [11]. Alginate is a natural biomaterial that exhibits excellent moisture absorption, biocompatibility, biodegradability, and nontoxic and flexible characteristics [115]. Alginate is also well known for its ability to activate the Nrf2 signaling pathway, increase the level of level of expression of antioxidant genes, reduce oxidative stress and inflammatory responses in cells, and promote cell regeneration and collagen synthesis, thus slowing the process of process of skin aging [116]. Consequently, alginate is an anti-aging biomaterial with great potential for use in various applications. In recent years, researchers have attempted to improve the properties (e.g., stability and permeability) of alginate via chemical and physical modifications, such as cross-linking, NP encapsulation, and other methods [117]. These modified alginate materials interact more effectively with the skin to exhibit an enhanced anti-aging effect. For example, Sobhanian et al. prepared an alginate nanofiber skin scaffold via the electrostatic spinning approach and observed good fibroblast viability and proliferation in the skin scaffold [118]. The combination of alginate with other antioxidants has also been attempted. Upon interaction with natural antioxidants, such as vitamins E and C, the antioxidant capacity of alginate is enhanced, leading to reduced free radical- and environmental-induced damage [119]. In one specific example, Busto et al. prepared an alginate film by cross-linking alginate with gellan gum, adding a polyphenolic olive extract as an antioxidant [120]. They demonstrated that this film not only reduced light-induced oxidative stress and inflammation, but it also helped restore normal fibroblast morphology and stimulated the expression of ECM proteins, ultimately slowing the photoaging process.

#### 4.1.3 Ribonucleic acid

Polydeoxyribonucleotide (PDRN) is a natural biomaterial produced by DNA degradation, which promotes cell growth and stimulates collagen and vascular endothelial growth factor production [121]. PDRN has been approved for tissue regeneration in Italy because of its biocompatibility, safety, low production cost, and lack of systemic toxicity [122]. In terms of skin anti-aging, some researchers have observed alterations in cell growth and proliferation, in addition to increased intracellular calcium ion and ECM protein production levels and decreased MMP13 concentrations after the PDRN treatment of embryonic stem cells obtained from healthy young donor skin [123]. PDRN has also been reported to play an equally prominent role in the proliferation and synthesis of fibroblasts [124], thereby indicating its potential to promote skin tissue regeneration. Consequently, a range of *in vivo*, *in vitro*, and clinical studies have been carried out to assess the effects of PDRN on several pathologies and in tissue regeneration using different methods of drug delivery [125]. For example, Shin et al. reported that PDRN promotes collagen accumulation in fibroblasts by promoting the synthesis of type III and type I collagen via the extracellular signal-regulated kinase (ERK) pathway [126]. The same research group also found that PDRN has anti-inflammatory effects as it inhibits the phosphorylation of ERK. In another study, Kim et al. deduced that PDRN inhibits tyrosinase activity by suppressing the expression of microphthalmia-associated transcription factor (MITF) and its target genes during melanogenesis, thus counteracting hyperpigmentation in aging skin [127]. Furthermore, Belletti et al. irradiated DNA-damaged fibroblasts with UVB radiation to establish a UVB damage model [128]. The subsequent application of PDRN to this system led to accelerated DNA repair along with a reduction in the number of cyclobutane pyrimidine dimers (with potential to cause DNA aberrations), γH2AX (a DNA breakage marker), and P53 (which is responsible for cell cycle arrest and apoptosis). These results demonstrate that PDRN also plays a positive role in reducing UVB-induced skin damage. These results therefore further reveal the basic role of PDRN in preventing skin aging and provide ideas for future clinical studies and the development of novel medical aesthetic products.

### 4.2 Synthetic molecular materials

PLA is a thermoplastic aliphatic polyester [129] that is considered one of the most promising biomaterials in the field of regenerative medicine due to its elasticity, rigidity, biocompatibility, thermoplasticity, and good molding ability [130]. In addition, PLA can be imparted with various functions upon the addition of different enzymes, antibiotics, natural compounds, metals, and metal oxides [131]. Based on its stereochemical structure, PLA can be classified as PLLA, poly DL-lactic acid, or poly D-lactic acid [131]; the most commonly used PLA in clinical anti-aging applications is PLLA. In human tissues, PLLA particles trigger a subclinical inflammatory response, which manifests as particle encapsulation, fibroplasia, and the deposition of type I collagen in the ECM. Over time, the inflammatory response wanes, PLLA degrades, and collagen accumulates [132]. The use of PLA microspheres with a diameter of 40–63 μm ensures that the particles are large enough to avoid phagocytosis by dermal macrophages and to prevent them from crossing the capillary wall; this size range also ensures that the particles are small enough to be injected with a fine 26-gauge needle [133]. Sculptra™ (Dermik Laboratories, Bridgewater, NJ), which is a product based on PLLA microspheres, has been widely used for injections into the cheeks, temples, subzygomatic area, chin, premaxillary sulcus, lifting line, infraorbital area, and lateral brow [134]. In addition, Kapicioğlu et al. reported that PLLA can effectively promote facial rejuvenation in a rabbit model, increasing dermal thickness and stimulating collagen production; these effects were retained even 6 months after application, thereby demonstrating the durability of PLLA in dermal filler applications [135]. Subsequently, in a randomized, controlled, multicenter, double-blind clinical study, Bohnert et al. found that PLLA stimulates the formation of neocollagen, which positively affects the physiological parameters of the skin, including the levels of hydration, elasticity, and trans-epidermal water loss (TEWL), along with overall skin quality (e.g., erythema, hyperpigmentation, pore size, radiance, and smoothness) [136]. In contrast, following the preparation of PLA NPs using a fibroblast monoculture and fibroblast–macrophage co-culture, Ray et al. [137] found that single fibroblast cultures did not promote collagen synthesis, while fibroblast–macrophage co-cultures appeared to be a good model for studying various cell–cell and biomaterial–cell interactions.

PCL is an aliphatic, semi-crystalline polymer that exhibits a rubbery consistency at physiological temperatures, endowing it with exceptional toughness and mechanical characteristics, such as a high strength and elasticity [138]. PCL can be degraded by microorganisms or by hydrolysis of its fatty ester chains to produce 6-hydroxyhexanoic acid, which is naturally metabolized in the body and is therefore safe [139]. Owing to the presence of five hydrophobic CH_2_ groups in its repeating units, PCL has a long degradation time and therefore possesses the longest duration of action among all polyesters [140]. In addition, PCL can be used to prepare block copolymers, where the amphiphilic properties as well as the mechanical and physical properties of the block copolymer can be improved by adjusting the ratio of the constituent blocks or by adding new blocks with the desired properties [141]. PCL is commonly used as a Class III medical device in the form of 25–50 μm microspheres for subcutaneous implantation in the face due to its excellent biodegradability and bioabsorbability, in addition to its ability to stimulate collagen regeneration [141]. Similar to PLLA, PCL fillers induce inflammation, proliferation, and collagen remodeling. During the remodeling phase, the early stages are dominated by granulation tissue formation and the appearance of type III collagen, followed by long-term production and deposition of type I collagen [142,143]. In addition, many fibroblasts, new elastic fibers, and capillaries have been detected close to the PCL microspheres. Research has also shown that PCL can be used for the long-term correction of facial wrinkles and other age-related facial conditions. For example, Ellanse™ (Sinclair Pharmaceuticals, London, UK) developed a filler consisting of 30 % synthetic PCL microspheres and 70 % carboxymethylcellulose (CMC) gel carriers, with a duration of action ranging from 9 months to 4 years [144]. Furthermore, FillerX™ (developed by GANA, Seongnam, Korea) extends the average maintenance time from 2 to 4 years by the addition of PCL microspheres to CLHA-based fillers [145]. However, one study found that patients treated with this filler may experience mild swelling and redness lasting 1–2 days, which may be caused by the multiple injection sites and/or by the glycerol present in the CMC gel [145]. To address this issue, Hong et al. developed a novel particle-free PCL filler, DLMR01, which is homogeneously soluble in water [64]. After evaluating its safety, biodegradability, and dermal collagen regeneration capacity, they demonstrated that particle-free DLMR01 PCL suspensions were effective in inducing neo-collagen formation and overcame the problems of uneven tissue regeneration and repair that existed with other dermal fillers. More recently, researchers have attempted to develop novel skin regenerating and anti-aging composites by combining PCL with other biomaterials. For example, Gautam et al. successfully developed a trimeric PCL/gelatin/collagen type I scaffold, which promoted fibroblast adhesion, proliferation, and differentiation, ultimately accelerating collagen regeneration and restoring skin fullness and elasticity [146].

### 4.3 Inorganic materials

Bioceramics are known to exhibit good biocompatibilities, high compressibility, and low tensile properties, in addition to promoting tissue regeneration and repair. Consequently, bioceramics have promising applications in regenerative medicine [147], with common materials including β-tricalcium phosphate and hydroxyapatite (HAp) [148,149]. Structurally, bioceramics are designed as three-dimensional (3D) porous scaffolds that offer mechanical support to tissues, withstand external pressures, preserve the integrity of the tissue form, and integrate with adjacent tissues to facilitate guided tissue growth [150,151]. Simultaneously, the porous structure promotes cell migration and growth, enhances nutrient and metabolite delivery, and promotes tissue integration and hemotransfusion reconstruction [152,153]. Studies have also suggested that some scaffolds are capable of releasing biologically active ions that promote the physiological behavior of cells and achieve therapeutic effects; therefore, bioceramics also show potential for application in the field of skin anti-aging [150,154]. For example, calcium HAp (CaHAp) fillers are commonly used to fill facial wrinkles, increase facial contours, and restore skin elasticity. CaHAp provides immediate filler results while stimulating collagen production, helping improve the overall skin texture and firmness. Consequently, the use of CaHAp (Radiesse, Merz Pharmaceuticals, Frankfurt, Germany) has increased in recent years. One of the main components of Radiesse's products is CaHAp microspheres (25–45 mm), which comprise 30 % of the final product, suspended in a 70 % gel carrier consisting of CMC, sterile water and glycerol. Following injection of the gel, the skin is immediately filled, and the gel gradually dissipates within a few weeks, while CaHAp remains at the injection site. After 3 months, the CaHAp microspheres at the site become encapsulated by a network of fibronectin, fibroblasts, and macrophages, and CaHAp acts as a scaffold for fibroproliferation and neocollagenesis. After 9 months, the microspheres begin to be absorbed by and detected in macrophages [155], thereby suggesting that the clinical effects of CaHA last for 12–18 months [156]. Consequently, bioceramics are often used as cosmetic implants in rhinoplasty, chin augmentation, and temple augmentation treatments, among others [157]. As implied by the above results, these implants can integrate with the surrounding tissues to enhance cosmetic results, while avoiding the problems associated with traditional filler materials, such as resorption, deformation, and displacement [158]. Moreover, the use of bioceramics has become more popular because of their good mechanical strengths and stabilities. In this context, Cai et al. designed a novel MN array on a flexible and self-expanding ceramic 10.13039/100006209NP substrate to combine structural support with drug delivery [159]. Upon contact with body fluids, the matrix separates from the needle, thus allowing the needle to remain in the skin, prolonging the release and effect of the drug for a longer period and improving the efficacy of the drug.

## 5 Role of biomaterials

### 5.1 Reduction of fine lines and wrinkles in the skin

#### 5.1.1 Mechanical tissue filling

The volume effect of a filler constitutes a key mechanism of action. More specifically, upon the injection of a biomaterial filler, a certain volume can be created inside the skin, filling wrinkles and depressed areas and helping the skin surface look fuller and smoother. This effect effectively improves the appearance of the skin to offer a more youthful and plumped facial contour [160]. The ideal tissue filler should have excellent biocompatibility, non-immunogenicity, and biodegradability, in addition to exhibiting desirable stability at the injection site and being cost-effective [80]. Currently, commonly used filler products include collagen, HA, polymethylmethacrylate, and autologous fat [161]. Of these, HA is most promoted in clinical practice and dominates the market because of its semi-permanence and safety. The most commonly used HA products on the market today are Restylane (Q-Med, Uppsala, Sweden), Hylaform (Sanofi, Paris, France), Juvederm (Allergan, Dublin, Ireland), and Captique (Allergan) [162]. All epidermal fillers are subjected to various combinations of compressive (transverse tensile) and transverse shear forces from both external and internal sources [163]. Therefore, the different anatomical levels of the skin require the selection of filler materials with different mechanical properties. For example, stiff fillers with a high modulus of elasticity are more suitable for deeper placement in the subcutaneous tissue or in front of the periosteum, which reduces the tactile feel of the gel particles. Softer fillers with a lower modulus of elasticity are often better suited for medium-to-superficial implants, such as in the correction of fine lines or skin folds [163]. Additionally, accurately assessing the individual characteristics of the patient and the injection site is important for the injector to develop a tailor-made treatment plan and achieve the best filler results [164]. For example, the deep layer of the superficial temporal fascia is a natural sliding plane, and the injector can smoothen the surface without irregularities by massaging the filler into recesses. The temporal region is rich in vascular nerves, and so the injector must use the cannula technique to reduce the risk of vascular injury [164,165]. In recent years, new dermal fillers have also been developed with the aim of providing superior stability and safety profiles. For example, Choi et al. developed an injectable levan hydrogel system and evaluated the potential of levan as a new material for dermal fillers [104]. More specifically, they prepared an injectable physical levan hydrogel by combining levan with CMC and polyphenolic compounds. *In vivo* results showed that these levan-based hydrogels have better biocompatibility and stability than HA-based hydrogels, and in a mouse wrinkle model, the anti-wrinkle effect and collagen content of the levan hydrogels were better than those of the HA hydrogels.

#### 5.1.2 Promotion of collagen synthesis

In recent years, various studies have demonstrated that biomaterials not only immediately increase the volume of the skin, making it plumper, but also stimulate the formation of new collagen. For example, HA injections cause the ECM to swell and exert mechanical tension on fibroblasts, which converts them into a stretched, active state, thereby inducing collagen secretion [101]. In this context, Quan et al. injected HA into the buttock skin of elderly patients and performed biopsies at different time points [166]. The results showed a significant increase in the content of type I collagen precursor (precollagen I), upregulation of TGF-β receptor expression, upregulation of growth factor expression, increase in epidermal thickness, and enhancement of TGF-β/Smad growth-related pathway gene expression in the injected skin. In addition, owing to their ability to trigger a subclinical foreign body inflammatory response, synthetic polymers (e.g., PLLA particles) can activate the inflammatory cells involved in the removal of foreign body particles and promotion of the repair process (e.g., neutrophils and macrophages). Such activation of the inflammatory cells is followed by fiber proliferation, which encapsulates the inflammatory particles and leads to the deposition of collagen [59]. In another study, Stein et al. investigated 21 women with a mean age of 56.9 years who were subcutaneously injected PLLA in the ventral forearm (a total of 1200 mg divided into four injections 3 months apart) [167]. Biopsies and genetic testing were performed between 2 weeks after the first injection and 10 months after the last injection. The results revealed an increase in type III and type I collagen production, as well as increased mRNA expression for TGF-β and higher levels of the MMP tissue inhibitor. After 28 months, the presence of PLLA particles was still observed, suggesting that PLLA degrades more slowly than previously reported. Overall, their study highlighted the long-lasting biostimulatory properties of PLLA on subclinical foreign body responses.

#### 5.1.3 Reduction in collagen degradation

As mentioned above, increased levels of ROS and inflammatory factors are mainly responsible for collagen degradation, skin laxity, and aging. Therefore, regulation of oxidative stress and inhibition of the inflammatory response are crucial for controlling skin aging [168]. In this context, some biomaterials are known to exhibit strong antioxidant and anti-inflammatory activities, including collagen, which is rich in various amino acids (e.g., glycine, hydroxyproline, and methionine) that can trap and neutralize free radicals to reduce cell damage [145]. In recent years, researchers have investigated the compositions of the specific antioxidant peptides present in collagen with the aim of targeting the extraction of antioxidant components to prepare new antioxidant biomaterials. For example, Wu et al. investigated the ability of salmon skin collagen hydrolysate to scavenge 2,2-diphenyl-1-picrylhydrazyl, hydroxyl radicals, and intracellular ROS in human umbilical vein endothelial cells [169]. Following purification with size exclusion chromatography, a new sequence peptide was identified, Pro-Met-Arg-Gly-Gly-Gly-Gly-Gly-Gly-Gly-Gly-Tyr-His-Tyr, which was determined using the oxygen radical absorbance capacity assay to exhibit a strong antioxidant activity. In addition to protein-based polymers, polysaccharide polymers can also act as anti-inflammatory and antioxidant agents. In this context, Fernando et al. found that HTT isolated from the edible brown alga *Sargassum maritima* not only inhibited ROS production in human dermal fibroblasts, but it also inhibited UVB-induced activation of NF-κB and MAPK signaling proteins and down-regulated pro-inflammatory cytokines (IL-1β, −6, −8, and −33 and TNF-α) [170]. Overall, this system was shown to exhibit a high anti-aging potential in the fight against collagen degradation. Biomaterials that do not possess anti-inflammatory and antioxidant activities can also be modified through structural modifications, the addition of antioxidants, control of their synthetic conditions, and the addition of natural extracts, thereby endowing them with anti-aging properties.

### 5.2 Reduction of pigmentation

Hyperpigmentation, which presents clinically as chloasma and age-related maculae, is one of the most important manifestations of skin aging [171]. Tyrosinase, one of the enzymes involved in pigmentation, is the central and rate-limiting enzyme in melanin synthesis. Tyrosinase plays a crucial role in the tyrosine metabolic pathway by catalyzing the conversion of tyrosine into dopamine oxidase, which promotes melanin production. More specifically, excessive tyrosinase activity leads to excessive melanin deposition, which in turn causes hyperpigmentation [172]. In addition, excess free radicals from oxidative stress can cause damage to melanocytes, leading to excessive melanin production and hyperpigmentation [14]. Therefore, to counteract the hyperpigmentation that occurs during skin aging, materials that exhibit anti-tyrosinase and antioxidant activities are essential.

Drug absorption through the skin is known to be challenging due to the protective barrier function of the umbilical cord layer. Therefore, researchers have attempted to improve the efficiency of transdermal drug delivery by using effective biomaterials [173]. For example, Yan et al. developed an HA MN to deliver the tyrosinase inhibitor glabridin, which was loaded with the antifibrinolytic agent tranexamic acid (exhibiting whitening properties) and an antioxidant vitamin C derivative (3-O-ethyl-L-ascorbic acid) [174]. They found that the growth of melanoma cells and the production of melanin were directly reduced in zebrafish following HA microneedling. In addition, Sparavigna et al. investigated the effects of combining HA with an anti-aging antioxidant complex consisting of vitamins, minerals and amino acids [175]. In their clinical trial, four female volunteers (37–60 years old) received four sessions of bio-regenerative treatment at three-week intervals. Multiple injections were administered to the face (outer corners of the eyes and cheeks), neck, nape of the neck, and dorsum of the hands. Consequently, both clinically and statistically significant improvements were detected in the complexion brightness and hyperpigmentation symptoms recorded for the participants.

Recent studies have also shown that some biomaterials themselves can inhibit pigmentation, without the requirement for incorporating additional drugs. For instance, Kim et al. investigated the effects of PDRNs on mitochondrial biogenesis, melanogenesis, and connective tissue proteins *in vitro* [127]. They found that PDRNs could inhibit melanogenesis by decreasing the expression of MITF and the activities of tyrosinase, tyrosinase-related protein 1, and tyrosinase-related protein 2. In another study, Chen et al. investigated the effects of natural seafood chitosan on melanin synthesis and melanosome transfer [176]. They found that chitosan effectively reduced basal melanogenesis and α-melanocyte-stimulating hormone-stimulated melanogenesis by inhibiting the expression of melanogenesis-related proteins and suppressing tyrosinase activity. The study also confirmed the inhibitory effect of chitosan on melanogenesis in human melanocytes.

### 5.3 Reparation of the skin barrier

The skin barrier is primarily located in the SC, which consists of keratinocytes surrounded by intercellular lipid sheets connected by the corneal stroma. Compared with the skin barriers of young people, older individuals exhibit slower keratinocyte renewal, an overall deficiency of key epidermal lipids (especially cholesterol), localized reduction in the SC intercellular film, and a decrease in the secretion of lamellar bodies. Ultimately, this results in a skin barrier that is more susceptible to damage, slower to repair, and more susceptible to environmental stimuli and injuries, which further exacerbates the sensitivity and fragility of the skin [177]. Therefore, enhancing the resistance of the skin barrier is necessary to slow aging. Recent findings show that by applying specific biomaterials, such as collagen hydrolysate and CLHA, the function of the skin barrier can be effectively enhanced to improve the protective and self-repairing abilities of the skin. In the elderly population, especially in individuals whose skin is prone to damage, dryness, and sensitivity, this approach of improving the skin barrier function is of great importance. For example, Shimizu et al. investigated the effects of the dietary collagens hydrolysates prolyl-hydroxyproline (PO) and hydroxyprolyl-glycine (OG) on skin barrier dysfunction in HR-1 hairless mice [178]. Their findings indicated that the intake of PO and OG led to a marked decrease in TEWL and significant enhancement in the water content of the SC. These outcomes imply that the administration of PO and OG could improve the compromised function of the skin's protective barrier. In another study, Sundaram et al. applied a standardized dose of HA to the surface of human skin explants and analyzed the water content of both the SC and whole epidermis, along with TEWL [179]. They found that CLHA demonstrated significant benefits in reducing TEWL, retaining and redistributing the intra-epidermal water, maintaining skin integrity, and improving the skin barrier structure and function. Further research and development of functional ingredients and products targeting the skin barrier are expected to provide new avenues for improving skin health and delaying aging.

## 6 Implant carriers for biomaterials

Different implant carriers can be used for the delivery of biomaterials in skin anti-aging therapies. The structural characteristics and key advantages of these carriers are presented in Table 1.

**Table 1:** Structural characteristics and key advantages of different carriers.

Carrier	Structural characteristics	Key advantages	Ref.
Microspheres	1. High volume-to-surface area ratio 2. Gaps between microspheres	1. Sufficient space for cell growth 2. Can promote cell proliferation and migration	[180,181,182]
Microneedles	1. Small size 2. Sharp head 3. Multi-channel design 4. Carrier design	1. Minimal nerve and blood vessel damage 2. Formation of micro-channels for smooth and comfortable drug delivery 3. Improved drug delivery efficiency 4. Easily fixed to the skin	[[183], [184], [185],^186^]
Hydrogels	1. Three-dimensional cross-linked structure 2. High water content 3. Tunable physicochemical properties	1. Good softness and elasticity 2. Morphological stability 3. Promote cell growth 4. Good moisturizing properties 5. Meet different tissue filling requirements	[187,188]
Nanoparticles	1. Large specific surface area and adjustable size 2. Nano-scale, small size 3. Able to form a protective film	1. Large contact area with the skin 2. Good penetration performance 3. Provide protection to antioxidants	[189,190,191,192]
Nanofibers	1. High porosity 2. Large specific surface area 3. Good mechanical properties	1. Provide sufficient space for cell growth and growth factor stimulation 2. Contact with a larger area of skin 3. Strong support for tissues	[[193], [194], [195], [196], [197], [198]]

### 6.1 Microspheres

**Fig. 6:**
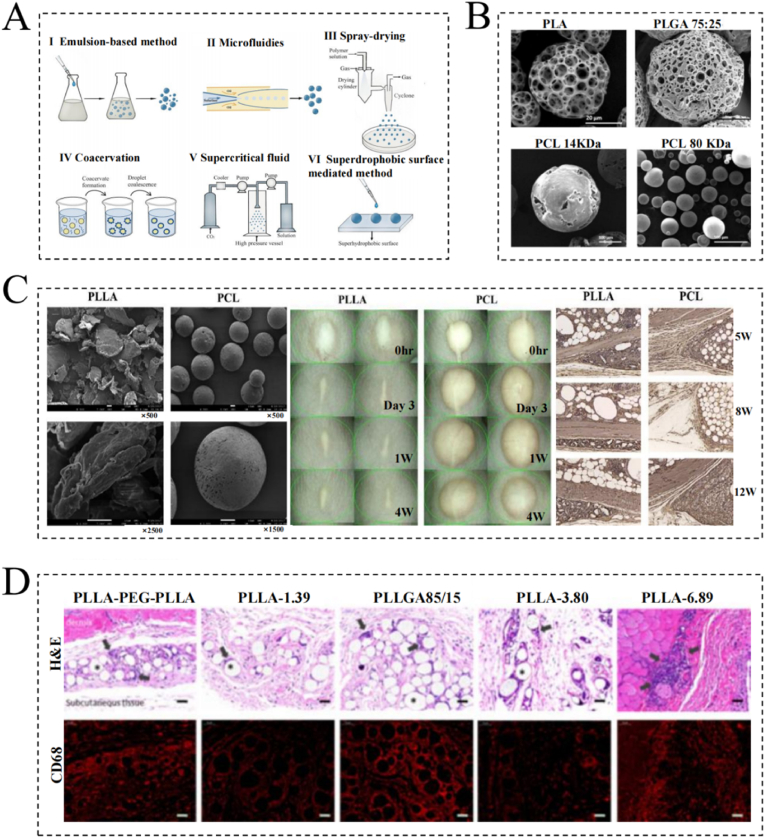
Microspheres. (A) Schematic diagram of the preparation process of microspheres. Reprinted from Ref. [199] with permission from Keai Publishing ltd. (B) Comparison of the morphology between porous microspheres and non-porous microspheres. Reprinted from Ref. [180] with permission from MDPI. (C) Scanning electron microscopy images of PLLA and PCL microspheres, changes occurring in the dorsal skin of hairless mice after implantation of microspheres, and histological manifestations of type I collagen deposition observed after implantation. Reprinted from Ref. [202] with permission from Wiley. (D) Electron microscopy photos of PLLA microspheres of different sizes/hematoxylin and eosin staining and immunofluorescence staining of microspheres prepared from different biomaterials implanted in the dermis or subdermal tissue. Reprinted from Ref. [181] with permission from Oxford University Press. Abbreviations: PCL, polycaprolactone; PLLA, poly (L-lactic acid).

Microspheres, which are also known as microcarriers, are injectable scaffolds. However, they differ from hydrogels in that they allow cells to adhere to and grow on their surfaces before injection into the defective area. Microspheres have a high surface area to volume ratio, providing enough space for cell growth. In addition, the gaps between the microspheres help promote cell migration and proliferation, thereby accelerating the formation of new tissue [180]. In general, microspheres measure between 1 and 1000 μm in diameter and should measure between 20 and 200 μm when used for injectable tissue engineering [199]. Examples of widely used microsphere-bound biomaterials are natural and synthetic biodegradable polymers [181]. Furthermore, in recent years, functional microspheres have attracted attention as novel injectable scaffolds for dermal fillers [200,201] owing to their ease of customization and fabrication, and as a result, the delivery efficiencies of cells and bioactive molecules have been efficiently enhanced (Fig. 6A and B) [180,199,201]. The main components of new-generation dermal fillers (e.g., SculptraVR) are PLLA microspheres, which can stimulate the regeneration of endogenous collagen and dermal fibroblasts after injection, as discussed above. This filler is effective in treating deep wrinkles or folds, and its results can be maintained for 18–25 months (Fig. 6C and D) [65,181,202]. In this context, Lemperle et al. evaluated the foreign body reaction induced by subcutaneous injection of New-fill (a dermal filler formulated with PLLA microspheres and marketed as Sculptra®) into the palmar region of the human forearm [203]. Observations showed the presence of PLLA microspheres in the soft tissue 2 weeks after injection. By 3 months, these microspheres were enveloped by macrophages and a few lymphocytes. After 6 months, the microspheres exhibited a porous or irregular surface and were encircled by macrophages. By the 9-month mark, the PLLA microspheres had seemingly “vanished” from the tissue. In another study, Goldberg et al. examined the tissue response to SculptraVR and found that the type I and type III collagen levels were significantly increased at 3 and 6 months after injection; the normal collagen levels were found to have increased after 12 months [204]. Furthermore, Zhang et al. explored the effects of different polymeric materials (i.e., different molecular weights, compositions, polymer structures, and conformations) on their biodegradation, inflammatory response, and collagen regeneration profiles [181]. Dermal filler degradation studies in rabbits have confirmed that these thermal fillers have good *in vivo* biodegradability and effective collagen regeneration. It was also shown that *de novo* collagen, especially type I collagen, was predominantly found around the microspheres and inside the fibrous capsules, whereas type III collagen was predominantly found outside the fibrous capsules. Moreover, a strong correlation was detected between the microsphere degradation rate and foreign body response, wherein a faster degradation rate led to a faster and significant inflammatory response. Consequently, fibrous capsules were generated more rapidly, and so they became less effective in maintaining the soft tissue volume.

### 6.2 Microneedles

**Fig. 7:**
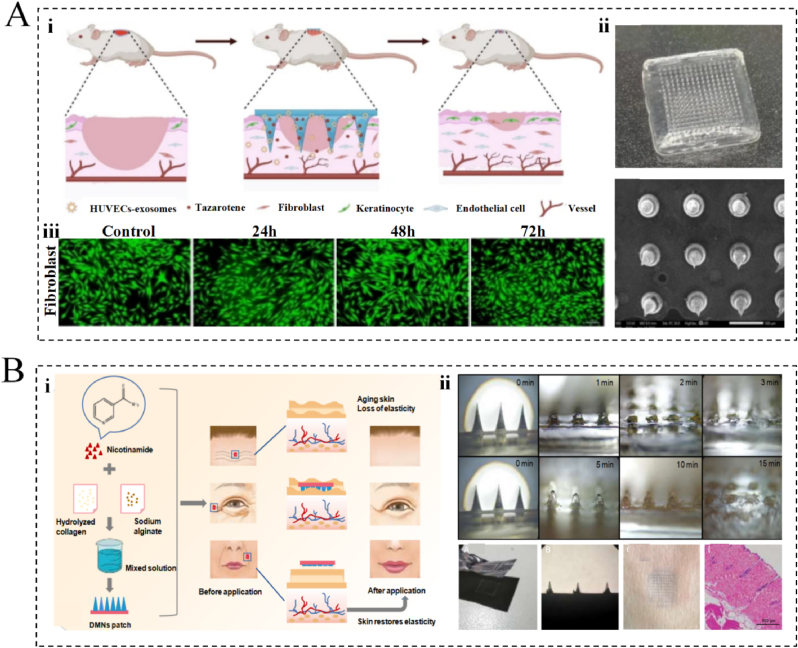
Microneedles. (A) Microneedle patches loaded with exosomes. (*ⅰ*) Schematic diagram of implantation in mice. (*ⅱ*) Extracellular matrix image of a microneedle patch. (*ⅲ*) Fluorescent staining image of skin after implantation. Reprinted from Ref. [205] with permission from BMC. (B) A novel anti-aging microneedle. (*ⅰ*) Schematic diagram of microneedle for anti-aging therapies. (*ⅱ*) Microneedle penetration and degradation ability. Reprinted from Ref. [207] with permission from Wiley.

MNs have received widespread attention as new, painless, noninvasive, and highly effective transdermal drug delivery systems (Fig. 7A) [205,206]. In contrast to other topical agents, MNs penetrate the skin to create microchannels, effectively bypassing the skin's protective barrier. This process facilitates the targeted delivery of medications to the epidermal and dermal layers while minimizing interactions with the nerves and avoiding blood vessel injuries [183]. The use of MNs simplifies the self-administration process for users and, being less invasive than traditional injections, may enhance patient compliance [184]. Morphologically, MNs consist of five main types, namely hollow, solid, coated, hydrogel, and dissolvable MNs (DMNs) [185]. DMNs have the advantages of simple production procedures, ease of use, and a high drug loading capacity. In addition, they are biocompatible and do not raise safety concerns. The tip dissolves completely in the skin tissue fluid and releases the drug at the same time (Fig. 7B) [207,186], rendering DMNs particularly suitable for cosmetic applications. Various investigations have been carried out into suitable materials for preparing MNs. For example, Lv et al. used HC as the main substrate and combined it with several commonly used biocompatible materials to overcome the poor mechanical strength of hydrocarbons alone [207]. In addition, they screened and prepared a flexible, skin-adaptable base DMN patch for collagen delivery. They also added niacinamide to MN to achieve the dual effect of reversing skin aging and treating skin diseases. In another study, Kang et al. applied a combination of traditional DMN patches and cream formulations to effectively deliver encapsulated drugs to the dermis [208].

### 6.3 Hydrogels

**Fig. 8:**
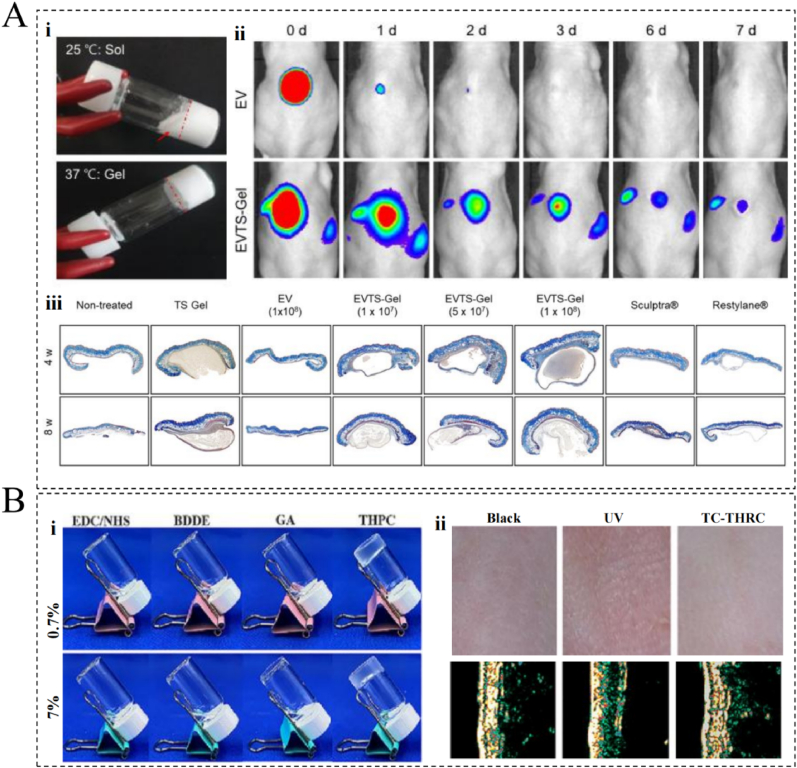
Hydrogels. (A) A stem cell-derived extracellular matrix injectable hydrogel for collagen production in the dermis. (*i*) Sol-gel transition images. (*ii)* Distribution of extracellular vesicles in untreated and hydrogel-treated mice. (*iii*) Effect of collagen production in mice *in vivo*. Reprinted from Ref. [211] with permission from ACS Publications. (B) Highly bioactive THPCcross-linked recombinant collagen hydrogel implant significantly improves skin quality after photoaging. (*i*) Degree of cross-linking. (*ii*) Skin microscopy and ultrasonic skin imaging after implantation in mice. Reprinted from Ref. [218] with permission from Elsevier. Abbreviations: Sol, solute; Gel, gelation; EV, extracellular vesicle; TS, thermosensitive; BDDE, 1,4-butanediol diglycidyl ether; EDC, 1-Ethyl-3-(3-dimethylaminopropyl)carbodiimide; GA, glutaraldehyde NHS,N-Hydroxysuccinimide; PEG, Polyethylene glycol; TC-THPC,THPC-crosslinked triplehelical recombinant collagen.

Hydrogels consist of 3D network structures bearing hydrophilic groups (e.g., NH_2_, COOH, and OH), which can absorb and swell in water. These 3D networks are typically composed of cross-linked polymer chains, such as those based on cross-linked colloidal clusters [187]. Owing to their high water content, soft structures, and excellent porosities, hydrogels can be considered similar to living tissue, exhibiting good water absorption and retention capacities, high elasticity, low toxicity, good biocompatibility, and desirable biodegradability. Consequently, hydrogels are considered to have outstanding potential for application in the field of skin tissue regeneration and skin anti-aging (Fig. 8A) [[209], [210], [211], [212]]. Although natural hydrogels, such as cellulose, chitosan, and collagen, are biocompatible, bioactive, and biodegradable, they generally exhibit poor stabilities and mechanical strengths. Moreover, although such natural hydrogels are generally safe, they can act as allergens, thereby presenting an immunological risk [213]. Synthetic hydrogels are prepared from synthetic polymers such as PCL, PLA, and hydroxyalkanoates, which impart them with good stability profiles and high mechanical strengths [214]. In contrast, semi-synthetic polymers represent a class of materials that combine the properties of natural polymers with those of synthetic ones through chemical modifications or amalgamation, as exemplified by methacryloyl-functionalized gelatin or acrylate-enhanced HA. Notably, these semi-synthetic hydrogels have shown promise in overcoming the limitations inherent in both natural and synthetic hydrogels within 3D cell culture systems, while effectively replicating the ECM of living tissues [215]. Therefore, the combination of natural and synthetic polymers has also become ideal for the preparation of semi-synthetic hydrogels. For example, gelatin methacryloyl (GelMA) is a semi-synthetic hydrogel obtained by reacting reactive amines and hydroxyl groups in gelatin with methacrylic anhydride (MAA), and by adjusting the amount of MAA, it is possible to control the degree of functionalization (DoF) of GelMA, which in turn affects its properties [216]. In addition, researchers have recently prepared hydrogels from polymers that are prominent in the field of anti-aging. For example, Busto et al. loaded olive leaf extracts rich in olive polyphenols into gellan gum/sodium alginate hydrogel films and conducted a comprehensive physicochemical characterization of the resulting composite hydrogels [120]. They also evaluated the delivery of olive polyphenols to the skin via the hydrogel film and found that the addition of *Olea europaea* L. leaf extracts to the hydrogel not only helped reduce skin damage from UVA exposure but also stimulated the expression of ECM proteins and helped restore normal fibroblast morphology. In another study, Sroka et al. designed a chitosan-based hydrogel containing extracts of *Vaccinium myrtillus* fruits and demonstrated the antioxidant, anti-hyaluronidase, and anti-tyrosinase properties of the gel [110]. Their results therefore suggested that the antioxidant properties of chitosan were retained, while the *V. myrtillus* extracts demonstrated a comparable anti-aging potential to that obtained from its topical formulations. In addition, Silva et al. produced and characterized a novel hybrid hydrogel of alginate mixed with elastin from bovine neck ligaments, which was found to promote the production of dermal fibroblasts [217]. Wang et al. developed a novel tetrakis (hydroxymethyl)phosphine chloride (THPC)-crosslinked recombinant collagen hydrogel for skin rejuvenation. Compared with traditional cross-linking agents, THPC achieves efficient cross-linking at low concentrations to form a hydrogel with high mechanical strength and stability. The hydrogel is biocompatible, promotes cell proliferation and migration, significantly improves dermal density and elasticity of photodamaged mouse skin, reduces water loss, improves SOD activity, and has anti-calcification properties (Fig. 8B) [218].

### 6.4 Nanoparticles

**Fig. 9:**
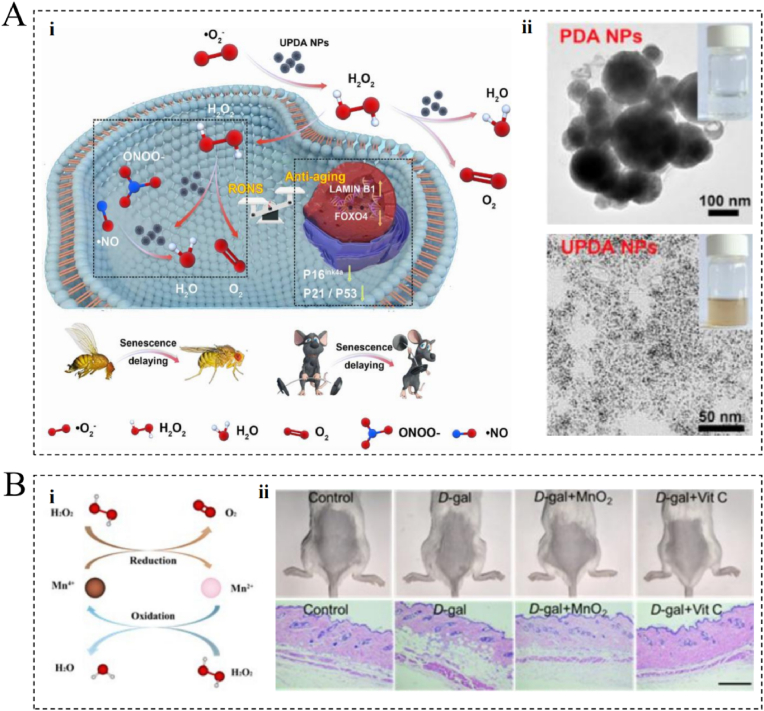
Nanoparticles. (A) Dopamine nanoparticles for anti-aging treatment. (*i*) Schematic representation of a nanoparticle antioxidant and anti-aging cells. (*ii*) Transmission electron microscopy images of PDA NPs and UPDA NPs. Reprinted from Ref. [190] with permission from Elsevier. (B) A manganese dioxide nanoparticle promotes skin anti-aging. (*i*) Schematic diagram of the reaction process of MnO2 NPs with H2O2. (*ii*) MnO2 NPs scavenge ROS *in vivo* in senescent mice and Masson's trichrome staining. Reprinted from Ref. [220] with permission from Wiley. Abbreviations: D-gal, D-galactose.

NPs are classified as particulate matter measuring <100 nm [189]. They are known to possess extremely high surface-to-volume ratios, in addition to specific functional groups that can attach other compounds to the surfaces of NPs or achieve targeted interactions with biological systems (Fig. 9A,9B) [219,190,220]. For example, natural polysaccharides are ideal NP materials for use in drug or protein delivery systems [221], with gelatin (a degradation product of collagen) and albumin also receiving widespread attention [222]. Furthermore, Schneider and Lim observed UV protection along with a skin-lightning effect in the case of ZnO NP coated with chitosan [223], while Aditya et al. reported that ZnO NP coated with peptides exhibit effective anti-inflammatory activity [224]. In terms of drug delivery, NPs are particularly desirable due to their large specific surface areas and highly adjustable sizes, providing a greater area of area of contact with skin cells to ensure effective drug delivery [191]. NPs also possess excellent skin penetration properties because of their small size, ease of penetration into the skin barrier, and direct delivery of antioxidants to the deeper layers of the skin. Such targeted delivery can significantly improve the absorption and utilization of antioxidants and enhance their effects on the skin [192]. Furthermore, NPs form a protective film that encapsulates the antioxidant, thereby protecting it from the environment (i.e., reaction with oxygen) and ensuring that it does not lose its activity during transport [225]. As a specific example, a clinical trial conducted by Felippi et al. was based on the encapsulation of active compounds (e.g., coenzyme Q10, retinyl palmitate, tocopherol acetate, and a mixture of grapeseed and flaxseed oils) in NPs to evaluate their safety and efficacy profiles [226]. They demonstrated that, in terms of safety, the NPs did not induce skin irritation, cytotoxicity, or oxidative stress and exposure of the NPs to UVA light did not result in photosensitivity. In terms of efficacy, subjects showed a significant reduction in the degree of wrinkles after 21 days of NP application compared to the control group, thus demonstrating the excellent potential of this system for application.

### 6.5 Nanofibers

Nanofibers are highly sought-after nanomaterials with many outstanding properties, such as large surface area-to-volume ratios, the possibility of surface functionalization, tunable porosities, a wide choice of materials, and excellent mechanical properties [193], which render them ideal for various biomedical applications. For example, to promote the growth of skin tissue, nanofiber scaffolds provide many of the properties required by cells for tissue regeneration and the sustained release of drugs or growth factors, such as a high porosity [194,195]. In addition, the large surface areas of nanofibers allow them to come into contact with greater numbers of cells to promote tissue growth [196]. Furthermore, their biodegradable nature permits their gradual degradation and absorption by the body without causing damage, while their excellent biocompatibility ensures the general absence of immune reactions [197]. In terms of mechanical properties, they can withstand the high strengths and pressures associated with mechanical extrusion, making them ideal as support materials [198]. Depending on the intended use of the fibers, different polymer materials can be employed, such as PLA, which can be used alone or with other polymers to produce copolymers. Owing to its excellent biodegradability and biocompatibility, PLA is widely used in the preparation of fiber materials in the biomedical field [227]. To further improve the stability and sustained drug release performance of PLA to ultimately reduce the required dosage, several PLA copolymers have been developed, including poly (L-propylidene-co-D,L-propylidene) and poly (lactic acid-co-α-glycolic acid), to prepare electrospun nanofibers [227]. In subsequent developments, biomaterial-based nanofibers were loaded with other antioxidants to combat oxidative stress and reduce the appearance of skin aging characteristics. For example, Sowmya et al. doped fenugreek into silk protein nanofibers and found that a higher fenugreek concentration led to enhanced antioxidant properties [228]. In another study, Berechet et al. added HC, a cowhide by-product, to ginger essential oil and produced bioactive nanofibers by means of electrostatic spinning [229]. They demonstrated that the antioxidant activity of the loaded nanofibers was significantly enhanced compared to that of the original HC.

## 7 Incorporation of other anti-aging substances

Other substances can be incorporated into skin anti-aging therapies. Table 2 presents a comparison of the advantages and disadvantages of different anti-aging substances and their combination with biomaterials.

**Table 2:** Comparison of the advantages and disadvantages of anti-aging substances and their combination with biomaterials.

Substance	Advantages	Disadvantages	Advantages of combining with biomaterials	Ref.
Stem cells	1 . Replacement of apoptotic and necrotic cells with healthy ones 2. Secretion of paracrine growth factors and cytokines 3. Release and activation of defensin peptides	1. Poor survival and differentiation rates 2. Difficulties in determining the best source of cells 3. Difficulty in managing stem cells from a clinical perspective	1. Mimicking the *in vivo* microenvironment 2. Providing adhesion sites for stem cells	[230,231,232]
Exosomes	1. Regulation of intercellular communication 2. Regulation of human skin fibroblasts (HDFs)	1. Expensive to isolate, purify, and mass produce; −80 °C storage and transport 2. Rapid clearance or poor retention during trauma 3. Limited absorption by the stratum corneum	1. Increased efficiency of drug delivery 2. Increased durability and stability of exosomes	[[233], [234], [235], [236], [237], [238]]
Growth factors	1. Induction of tissue regeneration	1. Limited safety profiles 2. Low cost-effectiveness 3. Lack of optimized delivery system	1. Improved growth factor stability 2. Targeted delivery of growth factors	[239,[240], [241], [242], [243],^244^]
Antioxidants	1. Retard and prevent oxidation of the cellular material	1. Unstable 2. Short duration of action	1. Individual antioxidant capacity 2. Enhanced antioxidant capacity of the antioxidant 3. Promoted penetration, absorption, and release of the antioxidants	[245,246]

### 7.1 Stem cells

Stem cells facilitate the rejuvenation of aging tissues and organs by replenishing aged or damaged cells with viable counterparts. Moreover, they exert anti-inflammatory and anti-apoptotic influences through the secretion of growth factors and cytokines, which act in a paracrine manner at the targeted therapeutic site [230]. In this context, Taub et al. recently demonstrated that specific stem cells present in hair follicles can produce new keratin-forming cells when activated by the defensin peptides released during neutrophil damage, further demonstrating their potential as a novel treatment approach for skin rejuvenation [230]. In addition, Zhong et al. found that dermal multipotent stem cells may stimulate the secretion and synthesis of collagen or elastin by fibroblasts through the activation of the P38/MAPK and TGF-b/Smad signaling pathways [247]. Consequently, growth of the ECM is promoted, eliminating wrinkles and enhancing skin elasticity.

While stem cell therapy presents significant promise and application potential, it faces constraints in clinical practice due to suboptimal survival and differentiation of grafted cells [231]. Additionally, the complexity of pinpointing the most suitable cell source, managing cell integrity, navigating the clinical nuances of stem cell handling, and clarifying their specific roles in well-defined clinical scenarios contribute to the challenges associated with the implementation of stem cell therapy [232]. Thus, the development of composite materials is of interest. For example, biomaterials can mimic the *in vivo* microenvironment through cell–substrate interactions, while also providing new means to regulate stem cells, providing adhesion sites for stem cells to maintain their strengths and properties. Biomaterials have also been reported to provide substrates for cell attachment, proliferation, functional differentiation, and migration [248]. In this context, Nowacki et al. injected a rat model with adipose-derived stem cells (ADSCs) combined with HA or ADSCs combined with fish collagen, using collagen alone and HA alone for comparison [249]. They found that the rats implanted with ADSCs exhibited higher mean filling effects and longer treatment durations than those observed in the control groups. No inflammatory reaction was observed in any of the groups, which confirms the safety and efficacy of fillers formulated with HA and ADSCs. Altman et al. investigated the potential of human ADSCs combined with non-animal stable HA as a novel injectable soft tissue filler [233]. Their work demonstrated that the combination of these stem cells with HA notably elevated the expression of pre-collagen-1 and -2 mRNA in a mouse model of thymic photoaging. Moreover, magnetic resonance imaging revealed that at the 3-week mark, the filler composed of ADSCs and non-animal stabilized HA provided consistent and enduring volume enhancement, accompanied by the development of a structured fibrovascular network. This finding led to the hypothesis that the injection of this combination could eliminate light-induced skin wrinkles in humans.

### 7.2 Exosomes

Aging fibroblasts produce greater quantities of ROS and MMPs, which ultimately leads to skin aging; however, exosomes can activate the MAPK, AKT, STAT3, and ERK1/2 signaling pathways to enhance the proliferation and migration of fibroblasts and simultaneously reverse UVB-induced aging [234]. In addition, exosomes can reduce the expression of MMPs and increase collagen and elastin production [235]. Despite such advances in this area, various challenges remain regarding the application of exosome therapy in a clinical setting. For example, the isolation, purification, and large-scale production of exosomes is costly, as are their transport and storage, which must be carried out at a low temperature of −80 °C [236]. In addition, exosomes can be rapidly cleared from the circulation and are poorly retained during trauma, thereby limiting their therapeutic efficacy [237]. Exosomes also possess limited penetration and absorption efficiencies in the SC [238]; the introduction of bioengineering techniques to exosome-based therapies is expected to solve these practical issues. In recent years, the combination of exosomal therapies with biomaterials has received increasing research attention, and these works have been aimed at increasing delivery efficiencies and producing slow-release therapeutic systems, which can ultimately maximize the therapeutic function of exosome scaffolds by increasing their durability and stability [250]. In this context, hydrogels have attracted attention because of their desirable multifunctional properties, as discussed earlier [251,252]. For example, Nooshabadi et al. investigated exosome-loaded chitosan–glycerol hydrogels as effective scaffolds for cutaneous wound dressings and skin tissue regeneration [253]. They found that this combination significantly improved exosome biocompatibility, providing compelling evidence for the development of innovative therapies based on exosomes and biomaterials. In a similar study, Liu et al. combined ADSC-derived exosomes with HA and applied their composite material in acute cutaneous wound healing in nude mice [254]. They found that this material significantly promoted fibroblast activity, re-epithelialization, and angiogenesis during wound healing, suggesting its role in promoting collagen regeneration and reducing wrinkle generation in aging skin.

### 7.3 Growth factors

Growth factors can interact with specific cell surface receptors and synergize with proteins to perform physiological functions that maintain skin health, wherein a reduction in growth factor levels plays a crucial role in skin aging [239]. Some studies have shown striking similarities between the oxidative events that promote skin aging and those that delay wound healing [255]. In both cases, the skin repair mechanisms are under great stress (e.g., reduced growth factor production), which leads to reduced levels of collagen and elastin, as well as a reduction in ECM regeneration [240]. Recently, several clinical studies carried out using human fibroblast-conditioned media have shown that when natural and physiologically balanced growth factors are topically applied to intact skin, they can reverse both the intrinsic and extrinsic signs of skin aging [241]. For instance, a clinical trial by Naughton et al. showed that growth factors can inhibit or reverse the process of cellular senescence in addition to stimulating ECM regeneration and promoting skin barrier function [239]. Nevertheless, their translation into clinical applications has been severely limited due to issues related to their safety and cost-effectiveness. For example, growth factors are used at the supraphysiological level without the incorporation of optimized delivery systems [242]. Such optimized delivery systems can enhance the therapeutic efficacy of growth factors by controlling the rate of growth factor release and regulating the chemical and physical properties of the delivery system, ultimately improving their stability and bioactivity *in vivo* and reducing the occurrence of side effects [243]. Concurrently, an optimized delivery system allows for the more targeted delivery of growth factors to specific tissue areas where they are required, avoiding non-specific distribution of the drug in the body, improving therapeutic efficacy, and reducing the required dosage [243]. Therefore, the combination of growth factors with biomaterials to provide optimized delivery systems has become a new focus for researchers. More specifically, when growth factors are combined with the ECM, the ECM releases growth factor signaling molecules at the required locations and under different kinetics depending on the cellular conditions [256]. In addition, the molecular complexes formed between growth factors and the ECM components modulate growth factor receptor signaling [257]. For example, recent studies have shown that glycosaminoglycan can modulate the activity of other proteins (e.g., growth factors and cytokines) by acting as a cofactor or limiting their bioavailability [258]. Lee et al. evaluated the anti-wrinkle effect of a preparation containing HA serum and human growth factor on wrinkles around the eyes (crow's feet) and showed a significant reduction in wrinkles after treatment [259]. Wang et al. compared a chitosan-cross-linked collagen sponge (CCCS) with a simple collagen sponge, and upon combination with recombinant human acidic fibroblast growth factor, they demonstrated that the CCCS-based system rapidly expressed greater quantities of TGF-β1 and collagen to promote skin tissue regeneration [260].

### 7.4 Antioxidants

The overproduction of ROS is known to significantly contribute to cellular senescence, apoptosis, and necrosis. During such processes, the affected cells become enlarged and express senescence-related markers, such as telomerase, alkaline β-galactosidase, 70-kDa heat shock protein, cyclin-dependent kinase inhibitor 2A (p16), and the tumor protein P53 [261]. Antioxidants play a key role in counteracting this process by slowing or stopping the oxidation of cellular materials, and to date, various natural compounds, such as herbs, spices, and phenolic extracts, have been found to exhibit significant antioxidant activities. These include gallic acid, catechins, eugenol, and various polyphenolic components of green tea extracts, such as EGCG. In addition, fruit extracts containing ascorbic acid (vitamin C), α-tocopherol (vitamin E), and β-carotene exhibit powerful antioxidant effects [245]. The application of these natural antioxidants may therefore provide novel strategies and approaches to slow the process of aging, reduce cellular damage, and promote cellular health. However, many antioxidants exhibit poor stabilities, thereby hindering their application and reducing their therapeutic effects [245]. To prevent physical and chemical damage to antioxidants, they have recently been combined with solid or semi-solid biomaterials [246]. Biomaterials not only interact with the antioxidants to enhance their antioxidant effects, but these materials also improve the penetration and stability profiles of the antioxidants in the skin, in addition to facilitating their release and absorption [246]. For example, Yang et al. combined curcumin with silk biomaterials and demonstrated that the interactions between stem cells and the surface-exposed curcumin significantly inhibited cellular senescence [262]. This inhibitory effect was confirmed by the downregulation of p16 and P53 gene expression and a reduction in β-gal staining, further suggesting that flavin-functionalized silk biomaterials open new avenues for numerous stem cell and tissue regeneration applications. In another study, Taskan et al. found that HA gels containing antioxidants (2 % HA, 1 % antioxidant, coenzyme Q10, or vitamin E and 5 % benzocaine) produced greater numbers of fibroblasts along with higher levels of type I and type III collagen [263]. Furthermore, Luo et al. incorporated three chemically diverse antioxidants, namely vitamin C, EGCG, and curcumin, into silk proteins and preserved them in phosphate buffered saline at pH 7.4 [264]. They found that strong interactions between the silk proteins and antioxidants exerted a significant stabilizing effect on the antioxidants. Moreover, Studzińska-Sroka et al. conducted a clinical study to assess the efficacy of injectable HA solutions and antioxidant complexes (minerals, vitamins and amino acids) in the treatment of skin aging and photoaging [110]. Clinically and statistically significant improvements were detected in terms of anti-aging efficacy, skin brightness, contour measurement parameters, deep skin hydration levels, and pigmentation.

## 8 Prospects and future work

This review summarized the mechanisms associated with skin aging and analyzed various natural and synthetic polymers, along with inorganic biomaterials, to determine their biological effects, possible modes of action, application strategies, and potential application in the field of skin anti-aging. However, many challenges remain in the application of biomaterials in this field, which can be broadly categorized into biological, technical, economic, and product development and regulatory aspects. Biomaterials must perfectly integrate with human tissues to eliminate potential immune rejection. They must also be biologically active and capable of stimulating cell growth and differentiation and of producing specific biological effects [265]. In terms of technological challenges, these mainly stem from the increasing demands on the performances of biomaterials, such as an improved biocompatibility, bioactivity, durability, and degradability. The processing and manufacturing technology of biomaterials is also a major challenge, as the material shape, size, and structure must meet the needs of any specific medical application [266]. Furthermore, the economic challenges are mainly derived from the high costs associated with the biomaterials themselves, which limits their widespread use in clinical practice [267]. In addition, the product development and regulatory challenges mainly refer to the fact that the development and application of biomaterials must adhere to a strict regulatory framework, including the selection of raw materials, control of the production process, testing and evaluation of the final product, and monitoring of potential side effects [268].Notably, various strategies are being investigated to overcome the above challenges. For example, the durability and degradability of a biomaterial can be improved by carefully designing and optimizing their physical and chemical properties, while their cost can be reduced through cooperation with governments, enterprises, and research institutes. Regulatory agencies, such as the US Food and Drug Administration (FDA) or the European Medicines Agency (EMA), have undertaken to develop a series of guidelines and standards to guide preclinical studies and clinical trials of biomaterials. In the future, with the advancement of science and technology, the application of biomaterials in the field of skin anti-aging will become increasingly extensive. Additional innovations are expected to emerge, bringing healthier and more youthful skin to humanity and injecting new vitality into the development of the medical aesthetic industry. Future research should focus on several key directions. First, the mechanisms of skin aging must be clarified to optimize the application of biomaterials. Second, novel types of biomaterials with stronger anti-aging effects should be developed, and clinical studies should be strengthened to validate the effectiveness and safety of such biomaterials. Such advances will be expected to achieve the goal of using biomaterials to fight skin aging in humans.

## CRediT authorship contribution statement

**Xin Dan:** Writing – original draft, Visualization. **Songjie Li:** Writing – review & editing, Visualization. **Han Chen:** Data curation. **Ping Xue:** Conceptualization. **Bo Liu:** Formal analysis. **Yikun Ju:** Methodology. **Lanjie Lei:** Supervision. **Yang Li:** Resources. **Xing Fan:** Funding acquisition.

## Declaration of competing interest

The authors declare that they have no known competing financial interests or personalrelationships that could have appeared to influence the work reported in this paper.

## Data Availability

No data was used for the research described in the article.

## Abbreviations

ADSCs: Adipose-derived stem cells
ECM: Extracellular matrix
ROS: Reactive oxygen species
NPs: Nanoparticles
UHMWPE: Ultra-high molecular weight polyethylene
PAAm: Polyacrylamide
DPBS: Dulbecco's phosphate-buffered saline
HClO: Hypochlorite acid
O2: Superoxide
DNA: Deoxyribonucleic acid
SASP: Senescence-associated secretory phenotype
LMNB1: Lamin B1
CAT: Catalase
GSH-PX: Glutathione peroxidase
GSH: Glutathione
D-gal: D-galactose
H2O2: Hydrogen peroxide
MDA: Malondialdehyde
TPL: Two-photon luminescence
UV: Ultraviolet
HA: Hyaluronic acid
PLA: Polylactic acid
PLLA: Poly-L-Lactic acid
PCL: Polycaprolactone
PG: Proteoglycans
BSA: Bovine serum albumin
Glu: Glutamate
Gly: Glycine
XPS: X-ray photoelectron spectroscopy
FTIR: Fourier-transform infrared
XRD: X-ray diffraction
JCPDS: Joint Committee on Powder Diffraction Standards
TEM: Transmission electron microscopy
DDR: DNA damage response
ATM: Ataxia telangiectasia mutated protein
ATR: ATR serine/threonine kinase
Chk1: Checkpoint kinase 1
MMP: Matrix metalloproteinase
MAPK: Mitogen-activated protein kinases
NF-κB: Nuclear factor kappa B
MNs: Microneedles
